# Comparative Characterization of Oxidative Enzymes for Arabinoxylan and Protein Cross-Linking via Ferulic Acid and Tyrosine in Model Systems

**DOI:** 10.1021/acs.jafc.5c09766

**Published:** 2025-12-07

**Authors:** Katharina Hoefler, Ulrich Sukop, Elisabeth Reiter, Denisse Bender, Mario Jekle, Patrik Roch, Margit Cichna-Markl, Stefano D’Amico, Regine Schoenlechner

**Affiliations:** † Institute for Animal Nutrition and Feed, AGES − Austrian Agency for Health and Food Safety, Spargelfeldstraße 191, 1220 Vienna, Austria; ‡ Department of Biotechnology and Food Science, 27270 BOKU University, Muthgasse 18, 1190 Vienna, Austria; § 26558 Department of Plant-based Foods, University of Hohenheim, Garbenstraße 25, 70599 Stuttgart, Germany; ∥ Department of Analytical Chemistry, University of Vienna − Faculty of Chemistry, Währinger Straße 38, 1090 Vienna, Austria

**Keywords:** biopolymer engineering, biocatalysis, phenolic compounds, oxidative coupling, buffer compatibility

## Abstract

This study comparatively investigated various oxidoreductases (laccase, peroxidase, tyrosinase, and glucose oxidase) and their combinations for their conversion capability of available connection points, tyrosine in proteins and ferulic acid (FA) in arabinoxylans, as useful cross-linking tools. Therefore, substrate specificity and pH- and temperature-dependent activity were studied in different substrate ratios. Enzyme characteristics varied notably across standard assays, where even substrate-dependent shifts in pH optima occurred. Combining enzymes significantly reduced the *K*_m_ for laccase with tyrosinase (0.504 to 0.238 mM) for tyrosine, whereas for FA, the *K*_m_ increased from 0.057 to 0.107 mM but decreased for peroxidase with glucose oxidase from 0.250 to 0.045 mM. The substrate ratio was found to be crucial to target homo- or heterocross-linking and the most effective simultaneous substrate conversion was reached by combining peroxidase with glucose oxidase at a 1:5 (FA:tyrosine) ratio. These outcomes provide valuable insights into the cross-linking behavior of oxidoreductases, supporting a rational selection for food structure improvements.

## Graphical abstract

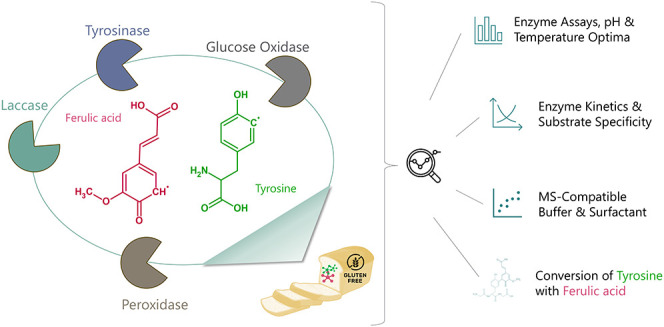

## 1 Introduction

Improving the nutritional properties and textural quality of food products remains a major objective in food research.^1^ A trend is the identification of ″clean label″ solutions, which renders enzymes during processing, a particularly valuable tool, since they are moreover a selective and environmentally friendly alternative.^1,2^ About one-third of enzymes are used in the baking industry, whereby cross-linking enzymes are the most common enzymes, which are mainly used in gluten-free (GF) bread formulations, to improve batter handling, rheological properties, and stabilizing the batter, resulting in improved bread properties.^1^ Common oxidoreductases include laccase, peroxidase, tyrosinase, pyranose oxidase, and glucose oxidase.^3^ These enzymes facilitate covalent cross-links between proteins and arabinoxylans (AXs), a major dietary fiber group in cereals, via oxidation of certain amino acid residues and phenolic moieties.^4–8^ For example, laccase was shown to enhance the volume and crumb structure in GF oat bread by improving gas retention during proofing.^9^ Further, tyrosinase and laccase were shown to affect the pore structure and volume in wheat bread, which were highly dependent on the enzyme type and dosage.^10^ Peroxidase, usually combined with H_2_O_2_ or glucose oxidase, was shown to improve dough handling by decreasing the adhesiveness through cross-linking AXs and proteins.^4,11^

Oxidoreductases vary in substrate specificity and catalytic mechanisms, leading to different degrees in inter- and intramolecular cross-links in AXs and proteins.^12^ These mechanisms involve further nonenzymatic reactions, contributing to the complexity of the processes, which is further dependent on both the used enzyme and the biopolymer. In detail, laccase, a polyphenol oxidase with four copper atoms in its active center, uses oxygen as a terminal electron acceptor to generate free radicals.^3^ Laccase demonstrates a broad substrate specificity, including in general any substance with a *p*-bisphenol structure, though tyrosine (Y) is considered a poor substrate.^13^ Peroxidases are a highly diverse group of oxidoreductases that utilize hydrogen peroxide as an electron acceptor, generating phenoxy radicals.^3^ These radicals can then undergo a coupling reaction, forming a quinone, and subsequently undergo nonenzymatic reactions resulting in complex structures.^13^ Peroxidases also exhibit a broad substrate spectrum, predominantly comprising aromatic compounds, including Y and ferulic acid (FA).^13^ Tyrosinases are type 3 copper proteins, containing a copper pair in the active center as electron shuttles, using molecular oxygen as an electron acceptor.^14^ Tyrosinase is a bifunctional enzyme that catalyzes the *ortho*-hydroxylation of monophenols and subsequently oxidizes diphenols to *o*-quinones. Subsequently, the *o*-quinones formed may undergo nonenzymatic reactions, resulting in complex cross-links via Y, cysteine, lysine, or FA residues.^3^ Glucose oxidases catalyze the reaction of glucose by consuming oxygen to generate a gluconolactone, which results in the formation of hydrogen peroxide, which is responsible for its cross-linking ability, which results in nonspecific cross-links.^3^ Pyranose oxidases, in contrast, exhibit a broader substrate specificity including mono- and oligosaccharides.^15^

Enzyme applications, despite their potential, still face challenges. The reported activity varies widely due to inconsistent assay conditions (e.g., substrate, pH, temperature), complicating dose comparisons and transfer of study results. For instance, excessive doses of enzymes can not only be costly but can also lead to no improvements or to undesirable effects.^16^ Moreover, the extent to which other substrates or enzymes affect enzyme activity remains uncertain. For e.g., peroxidase in combination with H_2_O_2_ was shown to increase the viscosity of wheat flour in higher concentrations, whereas lower concentrations showed the opposite result.^17^ Even the pH or temperature of a dough can significantly influence the activity and interaction of enzymes.^1^ So far, existing research already showed that for peroxidase, the AX to protein ratio is crucial, but data are still limited for other oxidoreductases; in addition, data on the effect of combining different oxidoreductases for a more sufficient cross-linking remain limited.^2,8^

A comparative characterization of various oxidoreductase activities sharing the same reaction conditions can establish a basis to model their cross-linking behavior of peptides or proteins with AXs. A common tool used for the identification of cross-linking products is mass spectrometry (MS).^4,18–20^ However, traditional phosphate buffers used in enzyme assays require additional preparation, such as desalting, to be compatible with MS.^21^ Using MS-compatible buffer systems simplifies the direct analysis of cross-linking products but can influence the enzyme activity.^22^ Additionally, many proteins and peptides have low water solubility, necessitating the use of MS-compatible solubilization agents such as detergents which do not inhibit enzyme activity.^23^ Thus, this work aimed to comparatively characterize the activity of commercially available oxidoreductases in MS-friendly model systems to provide a basis for a rational enzyme selection for developing strategies for food product structure enhancements. Thereby, laccase, peroxidase, tyrosinase, and glucose oxidases as well as their combinations were studied with different assays and the effect of homo- and heteroconversion of Y and FA was investigated in different ratios over time.

## 2 Materials and Methods

### 2.1 Materials

Tyrosinase from *Agaricus bisporus* (*AbT*) (EC 1.14.18.1) ≥1000 U/mg, laccase from *Trametes versicolor* (*TvL*) (EC 1.10.3.2) >0.5 U/mg, peroxidase from *horseradish* (*HrP*) (EC 1.11.1.7) type II 150–250 U/mg, glucose oxidase from *Aspergillus niger* (*AnG*) (EC 1.1.3.4) type X-S 100,000–250,000 U/mg, d-glucose ≥99.5%, 3-methyl-2-benzothiazolinone hydrazone hydrochloride monohydrate (MBTH) ≥99%, bovine serum albumin (BSA) ≥98%, l-tyrosine (Y) ≥99.0%, trans-ferulic acid (FA) ≥99.0%, and sodium periodate ≥99.8% were obtained from Sigma-Aldrich (St. Louis, Missouri). MS-grade ammonium acetate (AMAC), formic acid, and acetonitrile were purchased from VWR (Darmstadt, Germany). Ammonium bicarbonate (AMBIC) (MS grade), 2,2′-azino-bis­(3-ethylbenzothiazoline-6-sulfonic acid) diammonium salt (ABTS) ≥98%, and hydroxytyrosol (HTyr) ≥97.5% were obtained from Fisher Scientific (Schwerte, Germany). H_2_O_2_ (30%), potassium dihydrogen phosphate, citric acid monohydrate, and *N*,*N*-dimethylformamide (DMF) ≥99.9% (spectroscopy grade) were obtained from Merck (Darmstadt, Germany). RotiQuant 5*concentrate was obtained from Carl Roth (Karlsruhe, Germany), and RapiGest SF (MS grade) was obtained from Waters (Milford, Massachusetts). Ultrapure water (UHQ) was used for all analyses and was provided by an EnviroFALK (Westerburg, Germany) HALIOS system (conductibility <0.055 μS/cm and 0.22 μm filtered).

### 2.2 Enzyme Activity

Experiments were performed using a NanoQuant Infinite M200 PRO plate reader (Tecan, Maennerdorf, Switzerland) with the corresponding i-control software version 1.12.

#### 2.2.1 Molar Absorption Coefficient of HTyr-MBTH and ABTS

The volumetric activity (U/mL) of *AbT*, *TvL*, and *HrP* was determined spectrophotometrically by using HTyr-coupled assay with MBTH, which traps the enzyme-generated *o*-quinones to form a stable MBTH adduct with a high molar absorption coefficient.^24^ The molar absorption coefficient (ε) of HTyr-MBTH was determined in McIlvaine buffer (pH 4 to 6.5) by oxidizing 15–53 μM HTyr with the 5-fold concentration of NaIO_4_ for 20 min, followed by addition of MBTH (excess) and DMF (2%) to enhance the solubility of the HTyr-MBTH adduct, and the maximum absorbance was measured at a wavelength (λ) of 500 nm.^24–27^ The volumetric activity (U/mL) of *TvL*, *HrP*, and *AnG* was determined by using ABTS as the substrate. First, the ε of ABTS was determined in McIlvaine buffer (pH 4 to 6.5) by oxidizing 6–28 μM ABTS with *TvL* (2 mg/mL), and the maximum absorbance was measured at λ = 415 nm.

#### 2.2.2 Volumetric Enzyme Activity with HTyr-MBTH and ABTS

Activity measurements were performed in a reaction volume of 0.34 mL at 30 °C, and the absorbance was measured every 20 s for 3 min. For MBTH-HTyr, the reaction mixture contained 1 mM HTyr with an excess of MBTH and 2% DMF and 1 mM H_2_O_2_ in the case of *HrP*. The ABTS assay contained 1 mM ABTS, 1 mM H_2_O_2_ in the case of *HrP*, and 16 mM glucose and excess of *HrP* (0.7 mg/mL) in the case of *AnG* as coassay.^28^ Enzyme concentrations were adjusted to achieve an absorbance change of 0.1–0.2 per minute.

#### 2.2.3 pH and Temperature Optima

pH optima were tested in McIlvaine buffer between pH 4 and 6.5 (0.5 steps), and temperature optima were determined at the obtained pH optima at temperatures ranging from 25–50 °C (5 °C steps). The Michaelis constant (*K*_m_) and maximum reaction velocity (*V*_max_) were determined (at pH and temperature optima) by nonlinear curve fitting of six different substrate concentrations based on the Michaelis–Menten equation using the least-squares minimization method. The specific enzyme activity (U/mg) was calculated as the ratio of volumetric activity to protein content, which was determined via Bradford assay using bovine serum albumin (BSA) as the standard.^29^ All measurements were performed in triplicate.

### 2.3 Impact of Buffers and Solubilization Agents on Enzyme Activity

The impact of different buffers and surfactant concentrations on enzyme activity was tested with 0.1 and 0.05 M AMBIC (pH 6.5), 0.1 M AMAC (pH 4.5), and 0.1 and 0.05% RapiGest SF for *TvL*, *HrP*, and *AbT*. The total reaction volume was 1.5 mL containing 100 μM FA or 330 μM Y and 0.03 U/mL enzyme for FA or 0.01 U/mL for Y. Thereby, 1 U is defined as the change in absorbance of 0.01/min at 280 nm (Y) or 320 nm (FA), respectively, at pH 4.5 or 6.5, in AMAC or AMBIC buffer (0.1 M) at 30 °C in a reaction volume of 0.34 mL containing 33 μM FA or 167 μM Y. For *HrP*, additionally 1 mM H_2_O_2_ was added. Samples were incubated at 30 °C and the absorbance was measured every 20 s for 3 min at a λ_max_ of 320 nm for FA and a λ_max_ of 280 nm for Y.

### 2.4 Substrate Specificity for Tyrosine and Ferulic Acid

To ascertain substrate specificity, enzymes were incubated in McIlvaine buffer at pH 4.5 to 6.5 (0.5 unit steps) at 30 °C in a reaction volume of 0.34 mL with FA (2 mM) or Y (0.2 mM), respectively. The spectra were recorded between 230 and 800 nm in 5 nm steps at 30 s intervals (5 min) and after 30 and 60 min. Oxidation of FA resulted in a reduction of λ_max_ at 280 and 320 nm due to the loss of conjugation in the aromatic system. In respect to Y, an increase in λ_max_ at 280 nm due to the formation of dopaquinone was observed. Accordingly, the kinetic parameters *K*_m_ and *V*_ma_ (cf. chapter 2.2) were determined for FA and Y at six different substrate concentrations. For FA as substrate, the enzymes*TvL*, *HrP*, and the combinations of *AbT* with *TvL* and *HrP* with *AnG* were studied, using an ε value of 13,738 L mol^–1^ cm^–1^ at a λ_max_ of 320 nm.^30^ And for Y as substrate, the enzyme *AbT* and the combinations of *AbT* with *TvL* and *HrP* with *AnG* were studied, using an ε value of 5,375 L mol^–1^ cm^–1^ at a λ_max_ of 280 nm.^31^

### 2.5 Conversion of Tyrosine with Ferulic Acid Analyzed by HPLC-DAD

The reactivity of Y over time in the presence of FA was performed at different Y/FA ratios (1:1 or 5:1) for *TvL*, *HrP*, *TvL* with *AbT*, and *HrP* with *AnG* at pH 4.5 in 0.1 M AMAC buffer and for *HrP* and *AbT* at pH 6.5 in 0.1 M AMBIC buffer. All experiments were performed in a total reaction volume of 3 mL at 30 °C containing 33 μM FA and 33 or 167 μM Y and 0.03 U/mL enzyme. Thereby, 1 U is defined as the change in absorbance of 0.01/min at 280 nm (Y) or 320 nm (FA), respectively, at pH 4.5 or 6.5, in AMAC or AMBIC buffer (0.1 M) at 30 °C in a reaction volume of 0.34 mL containing 33 μM FA or 167 μM Y. One mM concentration of H_2_O_2_ was used in the reactions with *HrP* and 18 mM glucose with *AnG*. Immediately after enzyme addition and after 0.25, 0.5, 1, 2, 4, 6, and 24 h, 100 μL sample volumes were aliquoted and the reaction was stopped by the addition of 10 μL of 10% formic acid. Samples were vacuum-dried (1 h, 45 °C, >2000 Pa) using a Savant SpeedVac SPD 1030 vacuum concentrator (Thermo Fisher Scientific, Sunnyvale, CA) and stored at −20 °C. Prior to HPLC injection, the dried samples were resuspended in 200 μL of 10 mM KH_2_PO_4_ buffer (pH 2) containing 15% MeOH. A Chromaster HPLC system (Hitachi, Ibaraki, Japan) equipped with a Eurospher II RP C18 column (100 × 3 mm) and the corresponding guard column was used. A column temperature of 40 °C with gradient elution (conditions listed in Table S1) at a flow rate of 0.7 mL/min was applied. Quantification was performed by 9-point external calibration for FA and Y (0.1–18 μg/mL) for DAD at λ values of 280 and 320 nm. Peak areas were integrated using Chromaster System Manager software, version 1.2, from Hitachi.

### 2.6 Statistical Analysis

The statistical analysis was performed using GraphPad Prism version 9.4.0. Significant differences (*p* ≤ 0.05) were calculated by using the unpaired, two-tailed *t* test or ordinary one-way ANOVA with the Tukey posthoc test.

## 3 Results and Discussion

### 3.1 Enzyme Characterization and Suitability

The enzyme activities were characterized employing two different assays. Initially, the artificial and frequently used substrate ABTS was utilized, which can be converted by a wide range of enzymes.^32^ However, it is not a suitable substrate for *AbT*, and thus its use was limited to the determination of *HrP* and *TvL*. To better reflect enzyme activity under application-relevant conditions, a range of phenolic compounds was screened for their substrate specificity toward *TvL*, *HrP*, and *AbT*. HTyr demonstrated superior substrate specificity for all of the used enzymes (data not shown). As the reaction of HTyr to its *o*-quinone is neither stable nor suitable for sensitive detection, MBTH was utilized to trap the formed *o*-quinones, thereby forming a stable *o*-quinone-MBTH adduct, which is suitable for sensitive detection.^33^ The improved HTyr-MBTH assay provides a more realistic basis for the comparative characterization of the examined oxidoreductases due to the introduction of a phenolic group, compared to the widely used ABTS assay. However, to ensure a comparative analysis of the biochemical behavior of the enzymes, both assays were evaluated.

The ε values exhibited a linear relationship for ABTS within the concentration range of 6 to 28 μM at a λ_max_ of 415 nm, and for *o*-quinone-MBTH, within the concentration range of 14 to 53 μM HTyr at a λ_max_ of 500 nm, as illustrated in Figure 1. The ε values were determined over the pH range of 4 to 6.5 (0.5 unit steps). In this range, no changes in the absorbance of either ABTS or HTyr-MBTH were detectable (cf. Table S2). Accordingly, the mean ε values of 32,777 L mol^–1^ cm^–1^ for ABTS and 13,476 L mol^–1^ cm^–1^ for HTyr-MBTH were utilized for subsequent calculations. The ε of HTyr-MBTH has been described previously at a λ of 463 nm (the isosbestic point) with a higher ε of 22,000 M^–1^ cm^–1^.^33^ The deviation from the present value may be explained by the different wavelength used. For ABTS, ε values from 27,000 to 37,000 were reported. On the one hand, the mentioned values confirmed presented results, but showed a high variability as well.^34–36^ This complicates the comparability of the results obtained from enzyme assays. Consequently, the determination of ε remains a potential source of uncertainty in spectrophotometric assays, but determining ε under the actual assay conditions can minimize this source of error.

**Figure 1:**
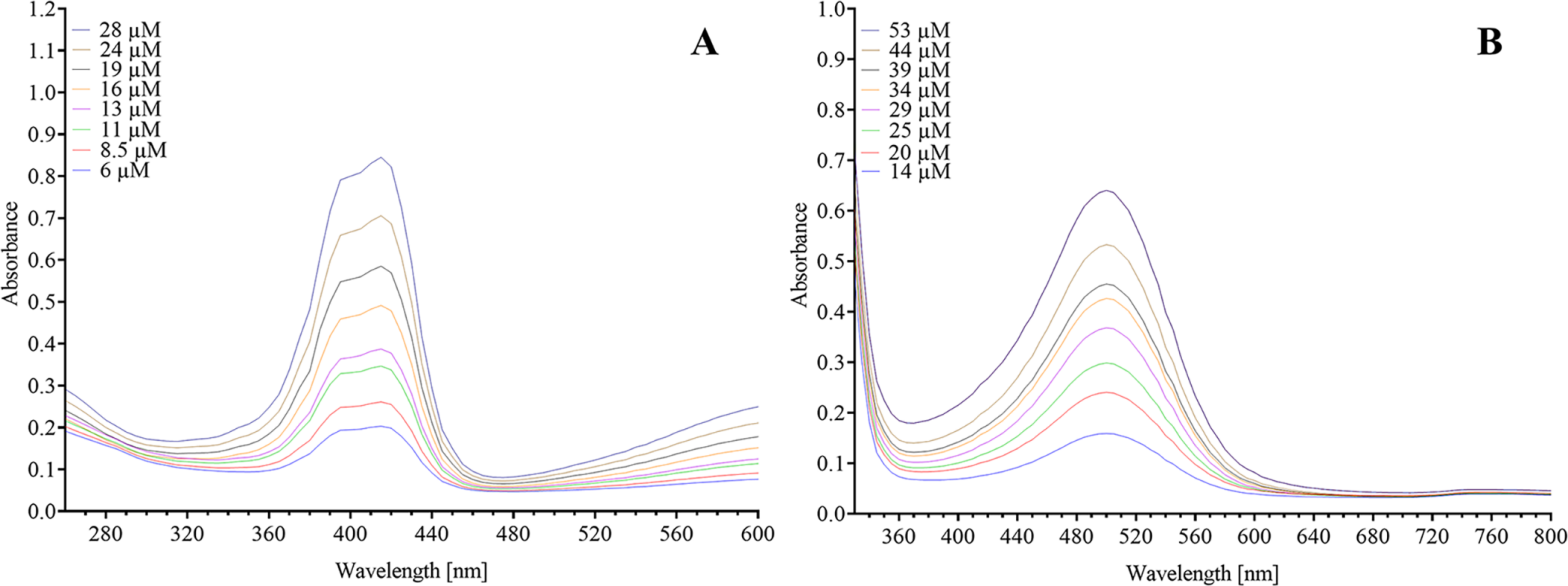
Exemplary absorbance product spectra from the oxidation of (A) ABTS with *TvL* and (B) hydroxytyrosol with NaIO_4_ as the MBTH-quinone adduct at pH 4.0 in McIlvaine buffer. Absorbance spectra are shown at different concentrations after reaching maximum absorbance.

In Figures 2 and 3 (cf. raw data, Tables S4 and S5), the pH- and temperature-dependent activities using ABTS and HTyr-MBTH assays are shown. Since the protein concentration varied from 0.2 to 50% (cf. Table S3), enzyme activities are given as specific enzyme activities to reduce the bias due to different product purities. As indicated in Figure 2, *TvL* performed best at lower pH values, with a monotonic pH profile for ABTS with the optimum at pH 4 and a bell-shaped profile for HTyr with the optimum at pH 4.5. These are in accordance with previous findings for fungal laccases, which showed that phenolic substances had a bell-shaped pH profile with optima around 4.5 and monotonic profiles for nonphenolic substances.^37,38^ The decrease in laccase activity at higher pH values is primarily due to inhibition by hydroxide anions, which bind to the T2/T3 copper centers.^37^ The fact that phenolic substrates show a biphasic pH profile with a slightly higher pH optima can be attributed to the increasing redox potential between phenolics and the T1 copper site at higher pH values.^37^ The balance between these two effects results in varying pH profiles for laccases, depending on the substrate.

**Figure 2:**
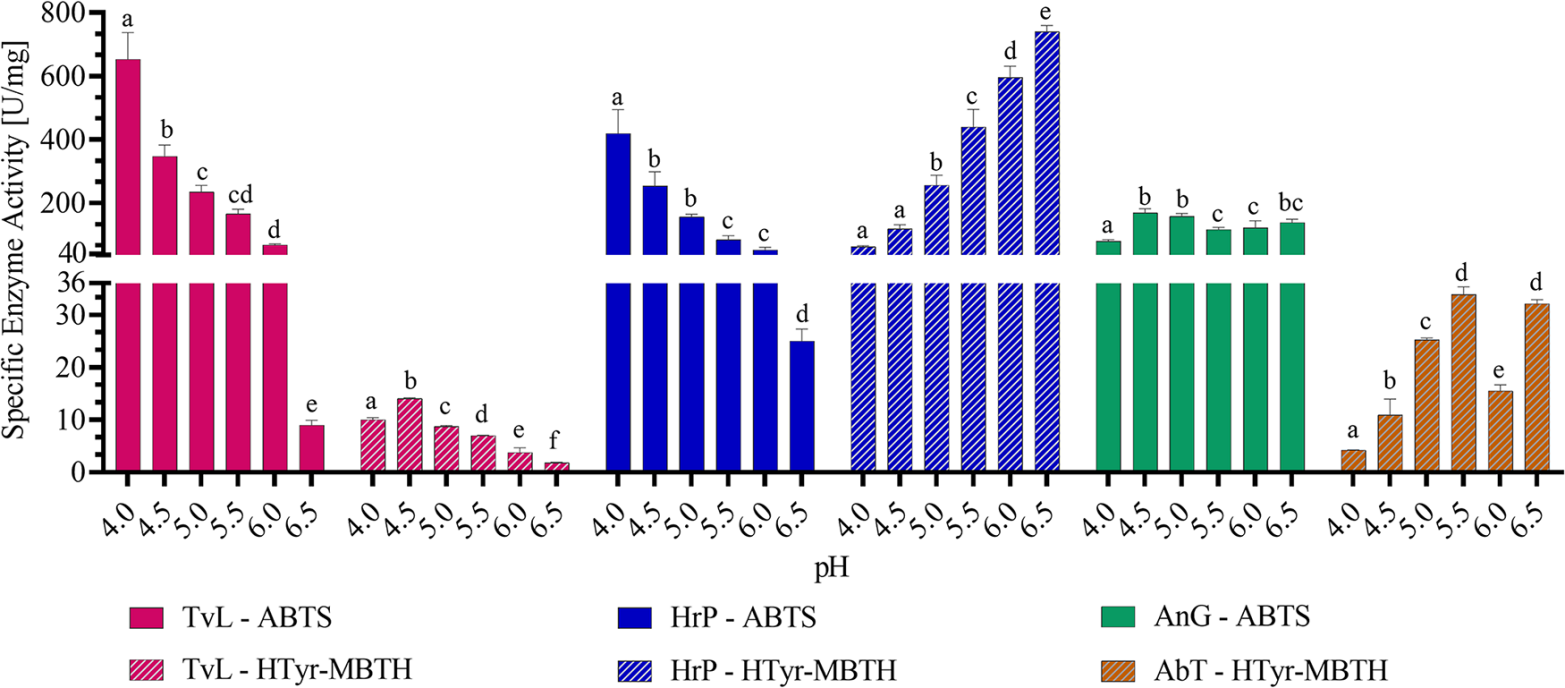
Effect of different pH values (4–6.5) on the specific enzyme activity of *TvL*, *HrP*, *AbT*, and *AnG* in McIlvaine buffer at 30 °C in a reaction volume of 0.34 mL. ABTS assay (*TvL*, *HrP*, *AnG*) contained 1 mM ABTS and in the case of *HrP*, 1 mM H_2_O_2_ and in the case of *AnG*, 16 mM glucose and excess of *HrP*, and absorbance change at 415 nm was monitored. Hydroxytyrosol (HTyr)-MBTH assay (*TvL*, *HrP*, *AbT*) contained 1 mM HTyr, excess of MBTH, and 2% DMF, and absorbance change at 500 nm was monitored. Results are shown as mean + SD (*n* = 3) and lower-case letters indicating significant differences (*p* ≤ 0.05).

**Figure 3:**
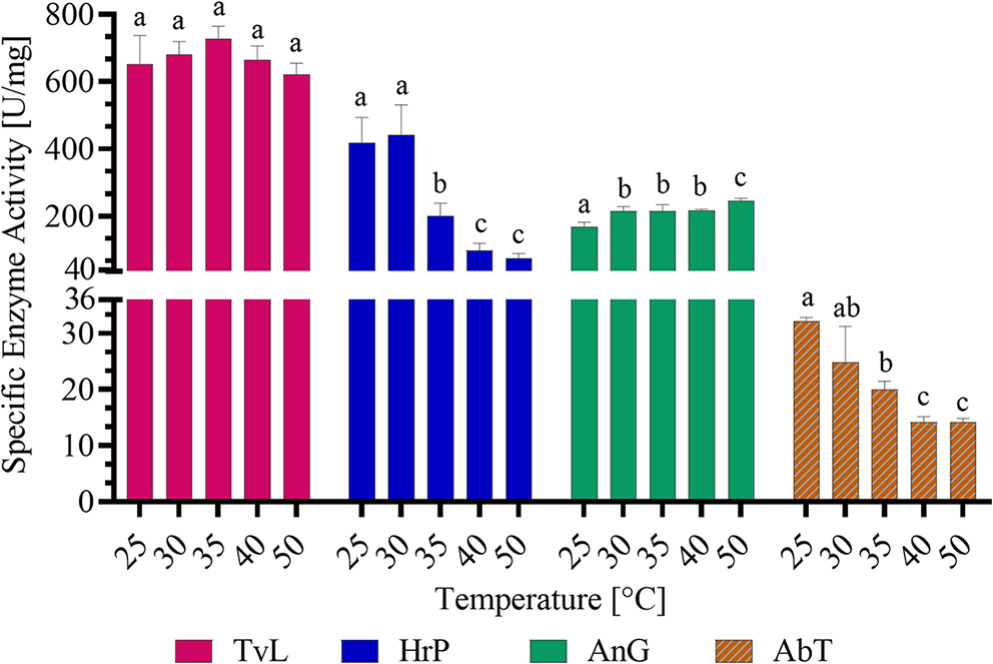
Effect of different temperatures (25–50 °C; 5 °C steps) on the specific enzyme activity of *TvL*, *HrP*, and *AnG* in McIlvaine buffer at pH 4.5 and for *AbT*, at pH 6.5 in a reaction volume of 0.34 mL. ABTS assay (*TvL*, *HrP*, *AnG*) contained 1 mM ABTS and in the case of *HrP*, 1 mM H_2_O_2_ and in the case of *AnG*, 16 mM glucose and excess of *HrP*, and absorbance change at 415 nm was monitored. Hydroxytyrosol (HTyr)-MBTH assay (*AbT*) contained 1 mM HTyr, excess of MBTH, and 2% DMF, and absorbance change at 500 nm was monitored. Results are shown as mean + SD (*n* = 3) and lower-case letters indicating significant differences (*p* ≤ 0.05).

For *HrP*, the monotonic pH profile for ABTS is similar to that for *TvL*, with the same pH optimum at pH 4, but showing a reverse monotonic trend for HTyr, with a pH optimum of 6.5 (Figure 2). This opposite behavior of *HrP* is strongly associated with the substrate, as already described by Hong et al.^38^ For the catalytic reaction of *HrP*, first H_2_O_2_ needs to react with iron­(III) as the electron donor to form compound I.^13^ This reactive intermediate can oxidize a substrate while forming compound II, a second active compound, which can oxidize a further substrate. Therefore, H_2_O_2_ affinity is crucial for the activation of the catalytic mechanism of *HrP*, but between the tested pH values, the affinity was shown to be constant with a *K*_m_ of 3.3 mmol/L.^39^ Thus, the observed differences are more strongly related to substrate-specific effects than to the activation of *HrP*. While the ABTS radical is more stable under acidic conditions, HTyr can be deprotonated at higher pH values (p*K*_a_ 9.45), explaining the distinct redox potentials leading to the reverse pH profiles of *HrP*.^40,41^ In contrast to *TvL* and *HrP*, *AnG* showed no significant difference with glucose as the substrate in the ABTS-*HrP*-coupled assay (Figure 2) from pH 4.5 to 6.5, which is in accordance with previous data and underline the applicability of *AnG* as a constant H_2_O_2_ supplier in different environments.^42^

For *AbT*, optima at pH 5.5 and 6.5 were observed, whereby a decrease at pH 6 was detected (Figure 2). While different pH optima have been described in the literature for *AbT*, bell-shaped pH profiles in the neutral to slightly acidic pH range are commonly observed.^24,43,44^ This was not detectable for *HrP* or *TvL* with the HTyr-MBTH assay, suggesting that solubility issues of the MBTH adduct or pH-specific changes to the substrate, rather than *AbT*, were the reasons for this observation. One possible explanation could be the reaction mechanism of *AbT*, which could lead to a decreased diphenolase activity through a conformation change at pH 6 or an increased polymerization of non-MBTH-trapped quinone.^24,27,45^ However, further research on pH-dependent structural changes of enzymes is necessary to test this hypothesis.

In general, temperature-dependent enzyme activity is based on a balance between two opposing factors. The increased activity at higher temperatures doubles approximately every 10 °C and the activity decreases with protein instability at higher temperatures.^46^ Therefore, depending on the temperature stability of the enzyme, different temperature-dependent activity curves can be detected. As shown in Figure 3, *HrP* and *AbT* showed a similar trend with an activity decrease from 25 to 50 °C, which is in accordance with previous findings that increasing temperatures are not favorable for these enzymes.^47,48^ *TvL* showed a broad temperature optimum, making it an easily applicable enzyme since the same amount of enzyme can be used over a broad temperature range without any necessary dosage adaption. *AnG*, which is known for its high-temperature stability, exhibited slightly increased activity at higher temperatures, reaching its maximum activity at 50 °C.^46,49^ Thus, *AnG* can be an effective H_2_O_2_ supplier over a broad temperature range. All enzymes showed sufficient enzyme activity at 30 °C, which facilitates a further comparison of oxidoreductases at this temperature.

The ability of *AbT*, *HrP*, *TvL*, and *AnG* to oxidize HTyr and ABTS was further evaluated by determining the kinetic parameters *K*_m_ and v_max_ (Table 1, corresponding enzyme activities cf. Table S6). The highest affinity for HTyr was observed for *AbT* (*K*_m_ 0.044 mM), which was slightly higher than that for *TvL* while being the lowest for *HrP* with a 7-fold increase in *K*_m_ (*K*_m_ 0.30 mM). The reversed order was observed for v_max_, whereby *HrP* (v_max_ 3014 U/mg) had a maximum reaction rate, which was about 300-fold higher compared to *AbT* (v_max_ 10.7 U/mg) or *TvL* (v_max_ 12.2 U/mg). It should be noted that a substantially higher v_max_ was observed for *HrP* in comparison to *TvL*, which contrasts with findings reported in previous studies. For instance, Xie et al. discovered that v_max_ is merely half of that of *TvL* for HTyr.^50^ However, this study was conducted under different pH and temperature conditions (pH 5, 50 °C compared to pH 6.5, 25 °C) and an alternative *HrP* source and detection method was utilized. Furthermore, 1 mM H_2_O_2_ was incubated with about 0.5 nmol of *HrP*, while in the study by Xie et al., 0.4 nmol of *HrP* was incubated with about 200 mM H_2_O_2_.^50^ Previous studies already showed that the H_2_O_2_ concentration is of crucial importance for the *HrP* conversion rate since at lower levels, a faster velocity could be achieved and at higher levels, the *HrP* species could become inactive.^51^ This observation underscores the critical influence that enzyme purity, source, and reaction conditions exert on the outcomes of kinetic value assessments, and thus it is crucial to identify comparable assays for the purpose of enzyme activity comparison.

**Table 1:** *K*_m_ (mM) and v_max_ (U/mL and U/mg) values of *AbT*, *TvL*, *HrP*, and *AnG* for HTyr-MBTH and ABTS Assays^a^,^b^

**enzyme**	**assay**	***K***_**m**_ **[mM]**	***V***_**max**_ **[U/mL]**	***V***_**max**_ **[U/mg]** ^c^
AbT	HTyr-MBTH^d^	0.044	1.69	10.7
***TvL***	HTyr-MBTH^d^	0.073	0.055	12.2
***TvL***	ABTS^e^	0.036	2.64	587
***HrP***	HTyr-MBTH^d^	0.30	1000	3014
***HrP***	ABTS^e^	0.0078	77.5	234
***AnG***	ABTS*-HrP*-coupled^e^	0.0021	303	265

a *AbT* = *Agaricus bisporus* tyrosinase; *TvL* = *Trametes versicolor* laccase; *HrP* = *Horseradish* peroxidase; *AnG* = *Aspergillus niger* glucose oxidase; HTyr = hydroxytyrosole; MBTH = 3-methyl-2-benzothiazolinone hydrazone hydrochloride monohydrate; ABTS = 2,2′-azino-bis­(3-ethylbenzothiazoline-6-sulfonic acid) diammonium salt.

b Kinetic values were determined at the corresponding pH and temperature optima by nonlinear curve fitting of six different substrate concentrations to the Michaelis–Menton equation by using the least-squares minimization method, the raw data of which are given in Table S6.

c Specific enzyme activity (v_max_ U/mg) was calculated as the ratio of volumetric activity (v_max_ U/mL) to protein content (Table S3).

d Absorbance change of product formation followed at 500 nm.

e Absorbance change of product formation followed at 415 nm.

In contrast, the highest affinity for ABTS was observed for *HrP* (*K*_m_ = 0.0078 mM), which was about five times lower for *TvL* (*K*_m_ = 0.036 mM). The v_max_ was comparable for both *HrP* (v_max_ 234 U/mg) and *TvL (*v_max_ 587 U/mg). Consequently, no correlation in the reaction kinetics between the different enzymes and assays could be identified. For *AnG*, glucose as a substrate was converted to H_2_O_2_ (cf. Figure S4B), which stoichiometrically activated *HrP*, leading to the radical formation of ABTS. Hereby, the lowest *K*_m_ values (*K*_m_ 0.0021 mM) were observed, compared to HTyr and ABTS as substrates for the other oxidoreductases, underlining the high affinity of *AnG* to glucose. The v_max_ of *AnG* (265 U/mg) was comparable to that of *HrP* with ABTS (234 U/mg), indicating the suitability of combining these enzymes.

The diverse trends in substrate affinities and reaction rates (Table 1), as well as the substrate-dependent pH optima (Figure 2), illustrate the complexity of comparing oxidoreductases using different assays. The observed differences can be attributed to diverse substrate types, which exert varying influences on enzyme catalysis. For instance, in the case of *TvL*, phenols have been observed to enhance the redox potential in the active center of the T1 copper atom, thereby increasing the enzyme activity at higher pH values. Furthermore, substrate-dependent activities were observed with *HrP*, attributable to the distinct binding characteristics of the substrate, including its electron density, size, and hydrophobicity, which is crucial for the binding of the enzyme–substrate complex and the electron transport of the substrate to the iron atom of *HrP*.^52^ No correlation was observed across enzymes or substrates, underlining the importance of carefully selecting and comparing assay conditions to enable a more accurate understanding of the optimal enzyme dosing. HTyr served as the substrate for three common and commercially available oxidoreductases *TvL*, *AbT*, and *HrP*. Coupled with MBTH, this is a simple and highly sensitive assay to identify the reaction differences between these enzymes.

### 3.2 Influence of Buffers and Solubilization Agents

Most enzyme assays use phosphate-based buffers since they are known for their broad buffer capacity, low UV absorption, and enzyme compatibility.^21^ For the identification of enzymatic cross-linking products, LC-MS with electron spray ionization (ESI) is a frequently used technique. However, phosphate-based buffer systems are not suitable for direct LC-MS measurements since phosphate salts can accumulate in the entrance cone of the MS and prevent sample ionization.^21^ Thus, the suitability of alternative MS-compatible buffer systems at optimal pH values of 4.5 for *TvL* and *HrP* and 6.5 for *AbT* and *HrP* (cf. Figure 2) was examined to avoid additional sample preparation steps in subsequent experiments.

For *TvL* and *HrP*, 0.1 M AMAC showed no significant effect on enzyme activity compared to 0.3 M McIlvaine buffer (Figure 4). In contrast, the *HrP* and *AbT* activities were significantly reduced in 0.1 M AMBIC buffer. Reducing the molarity of AMBIC to 0.05 M already restored the activity for *AbT* to McIlvaine buffer levels, indicating the strong influence of the AMBIC concentration. For *HrP*, even reducing to 0.025 M AMBIC only slightly improved the activity, but it was still about four times lower than that in McIlvaine buffer. As lower AMBIC concentrations would result in insufficient buffer capacity, they were not tested. Overall, ammonium ions (NH_4_^+^) seemed to have no significant influence on enzyme activity as well as acetic acid, which are the predominant species in AMAC buffer at pH 4.5. In contrast, hydrogen carbonate (HCO_3_^–^), the predominant species in AMBIC, might have reduced enzyme activities. However, since different substrates were used (pH 4.5 ABTS, pH 6.5 HTyr), HCO_3_^–^ or NH_4_^+^ may have affected the redox potential between the phenolic substrates and enzymes since the kinetic effects depend on electronic and steric characteristics of the substrate and hydrophobicity of the solvent.^53^ However, the effect was much stronger for *HrP* than for *AbT* and the solvent hydrophobicity did not differ significantly. Thus, it is more likely that the reduced activity is caused by enzyme inhibition due to HCO_3_^–^ rather than by the substrate oxidation potential or solvent hydrophobicity. Overall, 0.1 M AMAC and 0.05 M AMBIC are suitable MS-compatible buffers without a major enzyme activity impact, except for *HrP* at pH 6.5, where significant activity loss indicates unsuitability.

**Figure 4:**
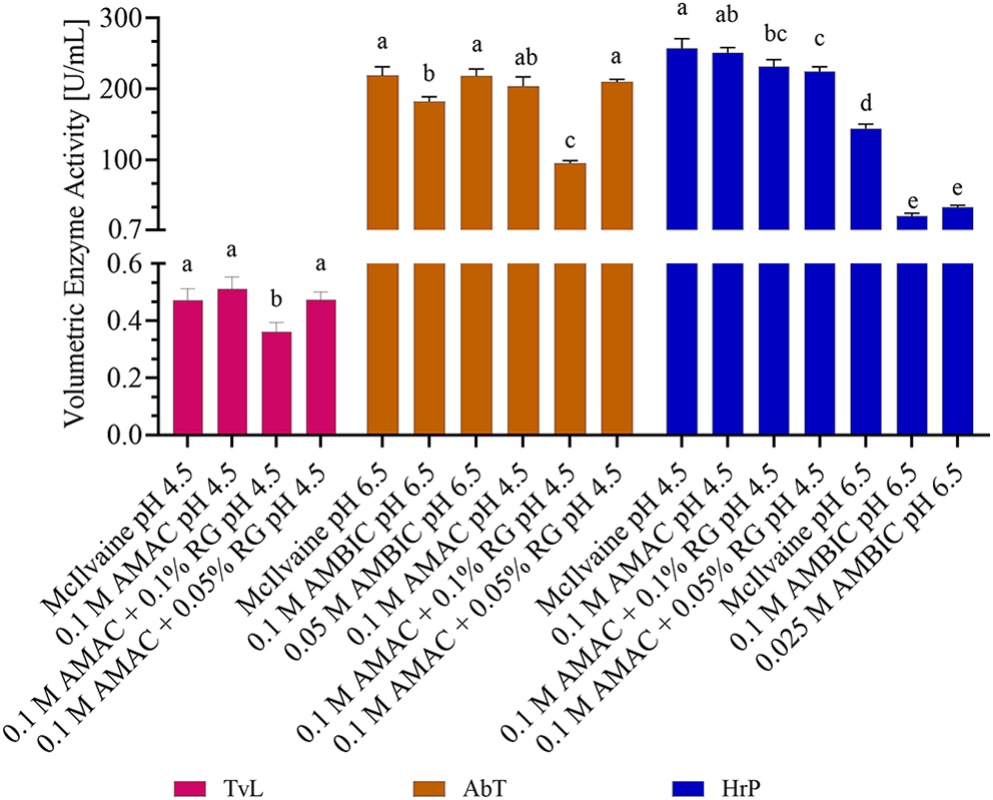
Effect of different buffers (McIlvaine, AMAC, AMBIC) and RapiGest SF surfactant (RG) as the solubilization agent at different concentrations on the volumetric enzyme activity of *TvL*, *HrP*, and *AbT* at 30 °C in a reaction volume of 1.5 mL containing 100 μM ferulic acid (*TvL, HrP*) or 330 μM tyrosine (*AbT*) with 0.03 U/mL enzyme for ferulic acid and 0.01 U/mL for tyrosine. In the case of *HrP*, 1 mM H_2_O_2_ was added to the reaction mixture. Results are shown as mean + SD (*n* = 3) and lower-case letters indicating significant differences (*p* ≤ 0.05).

Additionally, the effect of different concentrations of the MS-compatible anionic detergent RapiGest SF (RG) on enzyme activity was investigated, which can be directly used in a reaction system for cross-linking of poorly water-soluble peptides. RG enhances the solubilization and unfolding of proteins and is typically used to enhance proteolytic digestion prior to MS measurements.^54^ Since RG can be hydrolyzed with acids and the resulting byproducts have been shown to be compatible with MS systems, it was hypothesized that RG could be an excellent additive for enzymatic protein cross-linking.^54^ As can be seen in Figure 4 (cf. raw data Table S8), two RG concentrations (0.05 and 0.1%) in AMAC buffer were applied to *HrP*, *TvL*, and *AbT*. For all enzymes, a significant reduction in activity was observed with 0.1% RG compared to AMAC buffer. At 0.05%, no significant effects were observed for *TvL* and *AbT*. A marginal reduction in activity was observed for *HrP* (251 U/mL (AMAC) to 225 U/mL (AMAC + 0.05% RG)), which can be considered negligible. Accordingly, the addition of 0.05% RG is a MS-compatible approach, which was shown to be a suitable additive for enzymatic cross-linking for *TvL*, *HrP*, and *AbT*.

### 3.3 Enzymatic Conversion of Ferulic Acid and Tyrosine

The oxidoreductases were further evaluated, with respect to their conversion capability and reaction kinetics of the targeted cross-linking molecules in proteins and AXs. As shown in Figure S4, FA bound to arabinose residues on AX (Figure S4A1a) and Y in proteins (Figure S4C3a) are favorable candidates for cross-linking reactions, as they can undergo radicalization through enzyme-catalyzed processes (e.g., via *HrP* and *TvL* cf. Figure S4A,C), subsequently forming homocross-links with either FA (Figure S4D) or Y (Figure S4E) or heterocross-links (Figure S4F). Consequently, these two model substances are well suited for investigating the reactivity of the respective oxidoreductases, as the conversion of these phenols contributes to the probability of homo- or heterocross-link formation. Y is of particular interest due to its formation to quinones via *AbT* (Figure S4C). These quinones possess the capacity to form direct cross-links (Figure S4G–H) not only with Y or FA but also with other primary amines, such as lysine or cysteine, through further nonenzymatic reactions, such as Schiff’s base reaction or Michaelis addition, which results in further covalent protein bonds.

First, *TvL*, *HrP*, *AbT*, and *AnG* were incubated with FA or Y and the absorbance decrease at 320 nm (FA) or increase at 280 nm (Y) were monitored over 60 min. Based on the UV–vis spectra (Figure S1), the substrate specificities were shown to be quite different for the enzymes, and their conversion capabilities are summarized in Figure 5. For *TvL* (Figure S1B) and *HrP* (Figure S1D), FA was efficiently converted, whereby the proposed radicalization mechanism (Figure S4A) and the possible further homoreaction (Figure S4D) are shown in the Supporting data. For *AbT* (Figure S1F) and *AnG* (Figure S1H), only minor changes in absorbance were observed. Since FA underwent slow nonenzymatic oxidation over time as well, these minor changes were assumed to be caused by autoxidation.^55^ Thus, FA was only subjected to further kinetic analyses as with *TvL* and *HrP*, the results of which are shown in Table 2 (cf. enzyme activities in Table S7). *K*_m_ values were about five times lower for *TvL* than for *HrP*, confirming the high affinity of *TvL* to FA (*K*_m_ 0.057 mM), which is comparable to the HTyr kinetics (cf. Table 1). The v_max_ values were over ten times higher for *HrP* than for *TvL*. However, for *TvL*, the maximum reaction rate was about 20 times higher for FA (v_max_ 215 U/mg) than for HTyr (v_max_ 12.2 U/mg). In contrast, *HrP* showed comparable v_max_ values for both substrates (v_max_ FA 2512 U/mg and HTyr 3014 U/mg), underlining the less specific reaction mechanism of *HrP* (cf. Table 1).

**Figure 5:**
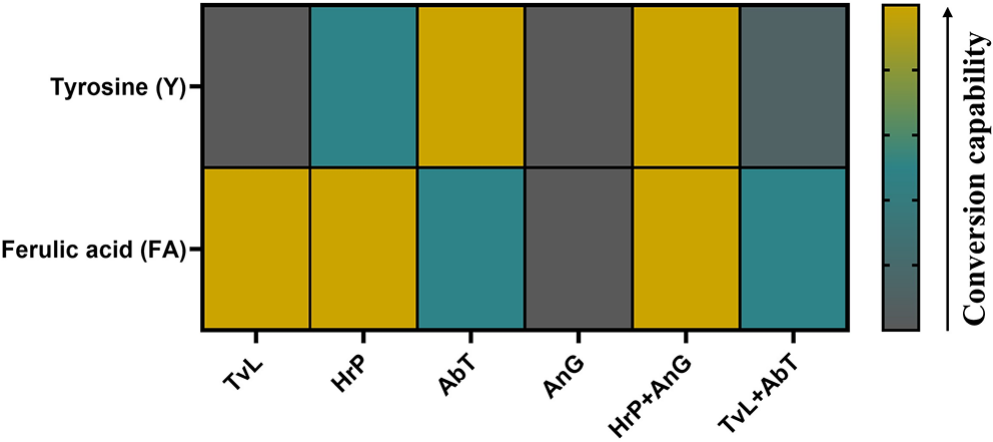
Heat map of the conversion capability of the oxidative enzymes *AbT*, *HrP*, *TvL*, *AnG*, and the combinations of *HrP* with *AnG* and *TvL* with *AnG* on tyrosine and ferulic acid. Values are based of the absorbance changes after incubation of the enzymes in McIlvaine buffer at pH 4.5 or 6.5 at 30 °C in a reaction volume of 0.34 mL with 2 mM ferulic acid or 0.2 mM tyrosine, respectively. The conversion capability increases thereby from gray over blue to yellow.

**Table 2:** *K*_m_ (mM) and v_max_ (U/mL and U/mg) values of *AbT*, *TvL*, *HrP*, *TvL* + *AbT*, and *HrP* + *AnG* for Tyrosine (Y) and Ferulic Acid (FA)^a^,^b^

**enzyme**	**substrate**	***K***_**m**_ **[mM]**	***V***_**max**_ **[U/mL]**	***V***_**max**_ **[U/mg]** ^c^
***AbT***	Y^d^	0.504	0.231	1.46
***TvL***	FA^e^	0.057	0.966	215
*AbT* **+** ***TvL***	Y^d^	0.238	0.081 (*AbT*)	0.511 (*AbT*)
			0.161 (*TvL*)	35.9 (*TvL*)
*AbT* **+** ***TvL***	FA^e^	0.107	0.228 (*AbT*)	1.44 (*AbT*)
			0.976 (*TvL*)	217 (*TvL*)
***HrP***	FA^e^	0.250	833	2512
***HrP*** **+** ***AnG***	FA^e^	0.045	1.64	4.94

a *AbT* = *Agaricus bisporus* tyrosinase; *TvL* = *Trametes versicolor* laccase; *HrP* = *Horseradish* peroxidase; *AnG* = *Aspergillus niger* glucose oxidase.

b Kinetic values were determined at the corresponding pH and temperature optima by nonlinear curve fitting of six different substrate concentrations to the Michaelis–Menton equation by using the least-squares minimization method, the raw data of which are given in Table S7.

c Specific enzyme activity (v_max_ U/mg) was calculated as the ratio of volumetric activity (v_max_ U/mL) to protein content (Table S3).

d Absorbance change of product formation followed at 280 nm.

e Absorbance change of substrate conversion followed at 320 nm.

As summarized in Figure 5, for Y, a high conversion was only observed for *AbT* (Figure S1E), with a strong increase at 475 nm, which indicates dopachrome formation, as shown in Figure S4C.^14^ *HrP* (Figure S1C) shows a moderate conversion, which was visible mainly through an increase in absorbance at 420 nm, which indicates dityrosine formation, whose radicalization mechanism (Figure S4C) and proposed further reaction (Figure S4E) are shown in the Supporting data.^56^ For *TvL* (Figure S1A), no significant change in absorbance was visible, while for *AnG* (Figure S1G), a small absorbance increase at 330 nm was observed, which could indicate the formation of an unspecific reaction product induced by H_2_O_2_. However, for *HrP*, no kinetic data for Y could be determined since the conversion rate over time was too low. Thus, for Y, only kinetic data for *AbT* was determined (Table 2). Here, *AbT* showed a ten times higher *K*_m_ value for Y (*K*_m_ 0.504 mM) compared to HTyr (*K*_m_ 0.044 mM) accompanied by a similar trend in the maximum reaction rate (cf. Table 1).

Additionally, two enzyme combinations, *HrP* with *AnG*, as a continuous H_2_O_2_ supplier (Figure S4B), and *TvL* with *AbT* were examined. Both were incubated with Y or FA up to 5 h, and the results are summarized in Figure 5 (spectra shown in Figure S2). A high conversion of both Y (Figure S2F) and FA (Figure S2E) was achieved when *HrP* was combined with *AnG*. These findings align with prior research that demonstrated the synergistic effects of *AnG* and *HrP* for cross-linking, e.g., proteins or pentosans.^57–59^ In addition, it has been shown that *HrP* activity increases with continuous H_2_O_2_ titration and was used as a technique for FA and Y cross-linking.^4,5,51,59^ This is primarily attributed to the mechanistic effect of *HrP*, whose catalytic activity is correlating with the H_2_O_2_ dose. At a low H_2_O_2_ concentration, *HrP* rapidly converts from the inactive form (*E*_0_) to the active form (*E*_2_), but only approximately 20% of the enzyme converts; thus, most of the enzyme remains in *E*_0_, limiting further enzyme activity.^51^ However, excessive initial H_2_O_2_ dosing results in the formation of an inactive *HrP* species, distinguished by the loss of the Fe atom.^51^ Thus, it is imperative that a continuous titration of low H_2_O_2_ doses is crucial for an effective *HrP* transformation to active compounds I and II, thereby ensuring a continuous high level of enzyme activity. As demonstrated by Schooneveld-Bergmans et al., the combination of *AnG* with *HrP* has been shown to result in equivalent cross-linking efficacy to that of continuous H_2_O_2_ titration.^59^ It is acknowledged that the absence of a control group in this study, with a continuous H_2_O_2_ titration (compared to *AnG* effect), prevents full exclusion of an enzyme–enzyme interaction, but based on the available literature, it is hypothesized that the observed synergistic effect is based on continuous H_2_O_2_ supply through *AnG*. For FA, however, no kinetic data could be determined since similar to *HrP* for Y, the conversion rate over time was too low (Table 2). The *K*_m_ values for the *HrP* combination with *AnG* were about six times lower (*K*_m_ 0.045 mM) than for *HrP* alone and even slightly lower than those for *TvL*, showing the highest affinity to FA of all examined enzymes. However, the maximum reaction rate of *HrP* with *AnG* was quite low (v_max_ 4.94 U/mg) and about 500 times lower compared to *HrP*. These results suggest that *HrP* with *AnG* can convert FA with high affinity in a slow and continuous way.

The combination of *TvL* and *AbT* was tested at two pH values, the respective optima of both enzymes. At pH 4.5 (Figure S2C), FA conversion was faster than that at pH 6.5 (Figure S2D), but the combination of the enzymes was much slower than that for *TvL* alone (Figure S1B). Therefore, the combination of *TvL* and *AbT* is counterproductive for the conversion of FA. For Y (Figure S2A,B), no significant change in absorbance was seen over 5 h at pH 4.5, and only a low change in absorbance was observed at pH 6.5. This indicates that a strong inhibition of *AbT* in combination with *TvL* occurs. Nonetheless, for both FA and Y, kinetic values could be determined (Table 2). When compared to *TvL*, *K*_m_ for FA increased with the enzyme combination (*K*_m_ 0.107 mM), whereas v_max_ showed no significant differences. However, for Y, compared to *AbT*, both *K*_m_ as well as v_max_ decreased (*K*_m_ 0.238 mM, v_max_ 0.511 U/mg). Thus, the combination of *AbT* and *TvL* changed the kinetic parameters for both Y and FA differently.

Given that the combination of enzymes was shown to affect substrate conversion, further investigations were carried out to determine whether the combination of substrates might modify the conversion rate. For instance, the presence of phenolic compounds such as FA has already been shown to change the conversion of other substrates through the transfer of radicals or as an antioxidant.^6^ Therefore, the conversions of FA and Y were subjected to further investigation when combined in a ratio of 1:1, as well as in a ratio of 1:5 (FA:Y), using the oxidoreductases alone and in their combinations. The conversion of Y with FA was observed over a 24 h period via HPLC-DAD measurements after incubation at pH 4.5 in AMAC and pH 6.5 in AMBIC buffer. The enzyme doses utilized were based on the change in absorbance per minute observed in the previously conducted investigations (Y at 280 nm with *AbT*; FA at 320 nm with *HrP*, *TvL*, and *HrP* with *AnG*). In addition, *HrP* was subjected to both pH values to detect possible differences of different buffer systems, and control samples were prepared in buffers without the enzyme but with H_2_O_2_ to observe effects of potential nonenzymatic substrate oxidation.

Given that pH exhibited a significant effect on enzyme activity (cf. Figure 2), it is crucial to note that the long-term pH stability (over 24 h) of the buffer was not evaluated. However, for the conversion of FA together with Y, long-term reactions were conducted. Consequently, the potential for some fluctuations over time (cf. Figure 6), attributable to minor pH instabilities, remains a subject of uncertainty. Nevertheless, it should be noted that this would have a considerable effect on the majority of the experiments. Thus, only the significant outcomes that were evident after 24 h are examined in greater detail in the following lines. As can be seen in Figure 6A,B, no significant influence through H_2_O_2_ oxidation could be observed. However, when FA was incubated in a 1:1 ratio with Y in both AMAC and AMBIC, a significant decrease in the FA concentration was observed (with and without H_2_O_2_). This indicates a nonenzymatic oxidation process of FA, which leads to a decrease of about 50% in AMBIC buffer and about 25% in AMAC buffer after 24 h (cf. Table S9). However, in a 1:5 ratio, autoxidation of FA could be prevented through the higher Y concentration. For Y, on the other hand, no significant changes in concentration were found in all control samples over 24 h.

**Figure 6:**
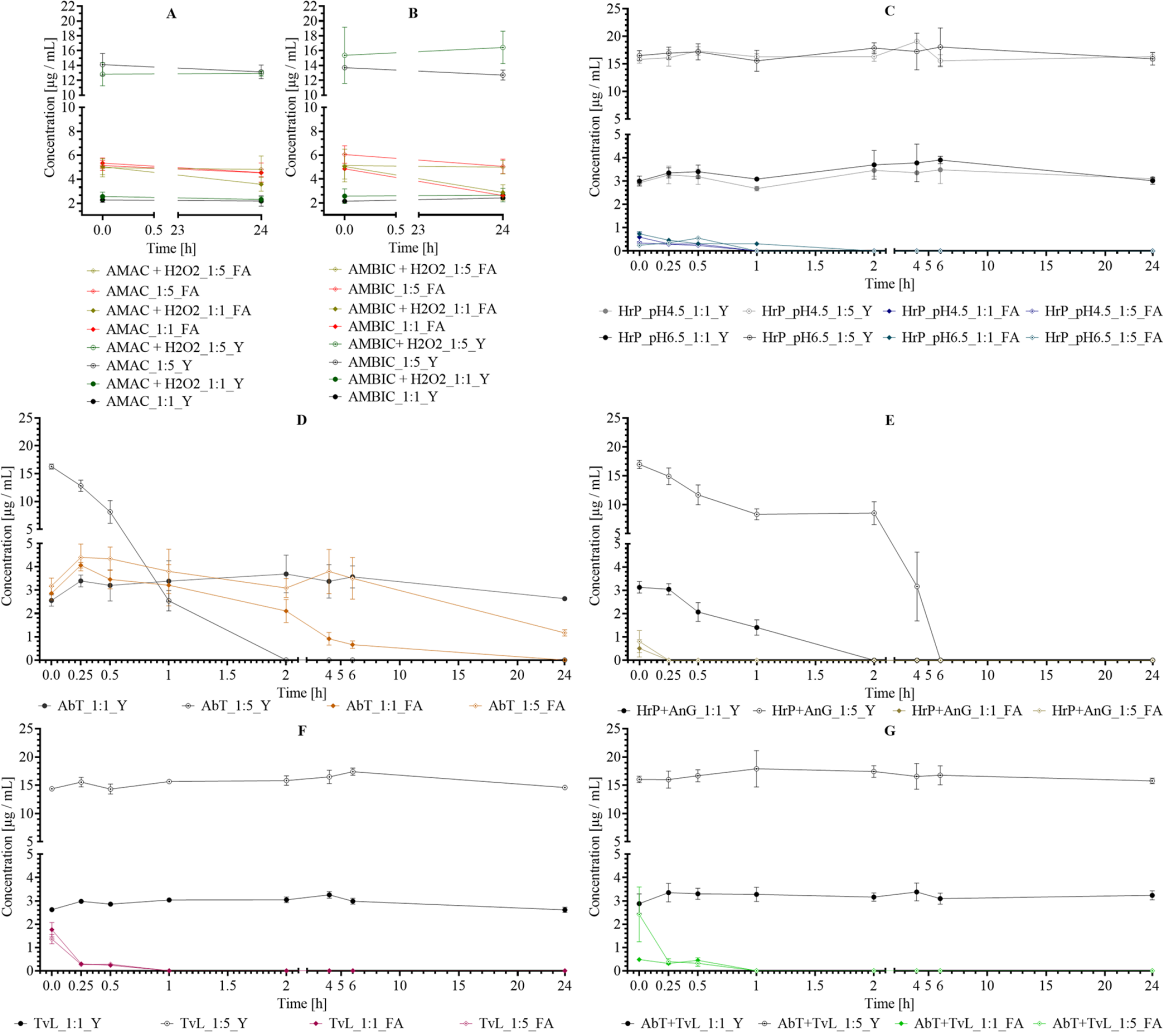
Conversion of tyrosine (Y) and ferulic acid (FA) over time (0 h: immediately after enzyme addition, 0.25, 0.5, 1, 2, 4, 6, and 24 h) in ratios 1:1 and 1:5 (FA:Y) for *TvL*, *HrP*, *TvL* with *AbT*, and *HrP* with *AnG* at pH 4.5 in 0.1 M AMAC buffer and for *HrP* and *AbT* at pH 6.5 in 0.1 M AMBIC buffer in a 3 mL reaction volume containing 0.03 U/mL enzyme. Results are shown as mean ± SD (*n* = 3), with (A) control samples in AMAC buffer and (B) for AMBIC buffer for FA and Y (ratio 1:1 and 1:5) with and without the addition of H_2_O_2_, respectively. (C) *HrP* at pH 4.5 and 6.5, (D) *AbT* at pH 6.5, (E) *HrP* with *AnG* at pH 6.5, (F) *TvL* at pH 4.5, and (G) *TvL* with *AbT* at pH 4.5 for FA and Y (ratios 1:1 and 1:5 (FA:Y)).

For *HrP* and *TvL* (Figure 6C,F), a similar substrate conversion behavior was observed, where the FA:Y ratio had no significant influence on the conversion. FA was fully converted after 1 h, which was in accordance with the conversion capability of FA without the addition of Y (cf., Figure 5). The pH values exhibited no significant disparities in the *HrP* conversion capacity, supporting the hypothesis that enzyme dosage adaption could be predicted with reasonable accuracy based on previously obtained data (Figure 6C). In contrast to incubation of Y alone with *HrP*, in the presence of FA, *HrP* did not convert Y as a substrate after 24 h (Figure 6C). But given that H_2_O_2_ was only added at the beginning of the experiment, it is likely that the entire H_2_O_2_ amount was consumed by *HrP* to convert the preferred substrate (FA), resulting in the subsequent inability of *HrP* to remain active after 1 h. This hypothesis was confirmed by combining *HrP* with *AnG* (Figure 6E). In this instance, the rate of FA conversion was also accelerated, but Y was subjected to conversion, as well, where a difference in the ratio was observed. At a 1:1 ratio, Y was found to be converted only after FA had been completely converted; thus, a homocross-linking of FA radicals (Figure S4D) is more likely, whereas when Y was added in higher quantities, it was converted immediately. This suggests that a higher Y concentration is required for heterocross-linking with *HrP* (Figure S4F). For *TvL*, even with a higher Y concentration, only FA homocross-linking of FA could be expected (Figure S4D).

The conversion capability of *AbT* (Figure 6D) showed significant differences in the examined substrate ratios. At a 1:5 ratio, Y was completely converted within 2 h, whereas at a 1:1 ratio, the Y concentration remained more or less constant. This might be explained by the competitive binding of FA to the active site of *AbT*, leading to inhibition.^60^ In detail, this phenomenon can be elucidated by examining the reaction mechanism of tyrosinases. Typically, tyrosinases exhibit a lag period in their monophenolase hydroxylation (Figure S4C), which is due to the reason that approximately 90% of the resting tyrosinase is in its *oxy*-form, a conformation that enables only monophenols to be catalyzed.^7^ Thus, the *oxy*-form must first be formed, which occurs indirectly from the *met*-form through the accumulation of diphenols.^7^ As Y, a monophenol, is the subject of this study, it is proposed that FA binding hinders this *met* to *oxy* conformation, thus forming a so-called dead-end complex, which explains why Y is not converted at a 1:1 ratio but with a higher Y ratio.^61^ However, FA could still function as a nucleophile and react with the formed quinones, leading to possible heterocross-links at a 1:5 ratio (FA:Y) as proposed in Figure S4H.^7^

In both ratios, FA was slowly decreasing over time, but since FA was previously not subjected as a substrate (cf. Figure 5), it is more likely that a slow autoxidation as found in AMBIC control samples took place (cf. Figure 6B). Thus, the use of *AbT* is preferred to build up homocross-links of Y (Figure S4G) but other nonenzymatic protein cross-linking reactions via primary amines (e.g., lysin or cysteine residues) can be expected as well (Figure S4G). However, due to the autoxidation of FA, which preferably happened at higher pH values, heterocross-linking products in minor amounts can also be expected with *AbT* (Figure S4H). The combination of *AbT* with *TvL* (Figure 6G), showed the same behavior as *TvL* alone (cf. Figure 6F), and even at a ratio of 1:5, *AbT* was not able to convert Y when combined with *TvL*. This is in accordance with the previous data from the experiments with Y alone (cf. Figure 5). Summing up, the combination of *TvL* and *AbT* did not exhibit an advantageous conversion behavior either for Y or for FA, individually or in combination. However, it is evident that nonenzymatic reactions constitute the predominant reaction pathway subsequent to enzymatic hydroxylation or radicalization, as demonstrated in Figure S4. Further, it has been ascertained that a significant proportion (approximately 50%) of FA undergoes nonenzymatic oxidation in AMBIC over a 24 h period (Table S9). Thus, the effect of enzymes is more purposeful in terms of the substrate-dependent reaction velocity since it can only provide a probability for subsequent reaction pathways, as the further nonenzymatic processes cannot be controlled. Thus, the available data only gives an estimation of the direction of the most probable processes.

The comparative characterization of oxidative enzymes as cross-linking tools revealed significant differences on the conversion of targeted substrates. Enzyme activity and kinetics varied notably between commonly used assays, with even pH optima shifting, depending on the substrate. The HTyr-MBTH assay was found to be suitable to compare the activities of *TvL*, *AbT*, and *HrP*, and the substrate specificity of FA and Y differed significantly among the enzymes. No benefit was observed from combining *TvL* and *AbT*, whereas *HrP* with *AnG* was the most effective for simultaneous FA and Y conversion. The FA:Y ratio was crucial to target homo- or heterocross-linking and the kinetic data of the targeted cross-linking molecules provide a basis to model the cross-linking behavior of theses enzymes. Moreover, it provides a solid foundation for the rational selection of oxidative enzymes in developing technologies to enhance the quality of food products. Nevertheless, only a model system with unbound substrates was applied here; thus, investigations on polymeric substrates need to be performed to verify the obtained results. Furthermore, it should be noted that the results of the rational enzyme selection are limited to the influence of buffer systems. Consequently, subsequent consideration should be given to the influence of complex food matrices on the enzyme performance.

## Supplementary Material



## Data Availability

Supporting Data are available in the appendix.

## Notes

Chromatographic gradient conditions for HPLC-DAD measurements of tyrosine and ferulic acid conversion according to chapter 2.5 (Table S1); determined molar extinction coefficients with the corresponding R^2^ of ABTS and HTyr-MBTH at different pH values (Table S2); list of protein concentrations of the used enzymes (*TvL, HrP, AbT*, and *AnG*) determined via Bradford assay (Table S3); list of the corresponding raw data of the pH-dependent specific enzyme activities as shown in Figure 2 for ABTS and HTyr-MBTH assays (Table S4); list of the corresponding raw data of the temperature-dependent specific enzyme activities as shown in Figure 3 using ABTS and HTyr-MBTH assays (Table S5); list of raw data of the volumetric enzyme activities of enzyme kinetic studies with ABTS and HTyr-MBTH assays as shown in Table 1 (Table S6); list of raw data of the volumetric enzyme activities of enzyme kinetic studies using FA and Y as shown in Table 2 (Table S7); list of raw data of the influence of buffers (AMAC, AMBIC, McIlvain) and different surfactant concentrations on the volumetric enzyme activity as shown in Figure 4 (Table S8); list of raw data of FA and Y concentration change after conversion in different ratios (1:1 and 1:5) over 24 h incubation analyzed via HPLC-DAD as illustrated in Figure 6 (Table S9); UV–vis spectra of conversion capability over 60 min of Y and FA through *TvL, HrP, AbT*, and *AnG* (Figure S1); UV–vis spectra of conversion capability over 5 h of Y and FA through enzyme combinations of *AbT* with *TvL* and *HrP* with *AnG* at different pH values (Figure S2); regression lines with the corresponding equation and R^2^ for FA and Y HPLC-DAD measurements, corresponding to method 2.5, and an example of a typical chromatogram showing Y and FA retention times (Figure S3); and selection of the proposed reaction mechanism of *TvL*-*, HrP*-*, AbT*-, and *AnG*-catalyzed reactions (Y, FA, and glucose), whereby further possible nonenzymatic reactions of the enzyme-catalyzed reaction products (homo- and heterocross-linking structures) are shown (Figure S4) (PDF)

## Abbreviations

AbT: *Agaricus bisporus* tyrosinase
ABTS: 2,2′-Azino-bis­(3-ethylbenzothiazoline-6-sulfonic acid) diammonium salt
AMAC: ammonium acetate
AMBIC: ammonium bicarbonate
*AnG*: *Aspergillus niger* glucose oxidase
AX: arabinoxylan
BSA: bovine serum albumin
DMF: *N*,*N*-dimethylformamide
FA: ferulic acid
*HrP*: *Horseradish* peroxidase
HTyr: hydroxytyrosol
MBTH: 3-methyl-2-benzothiazolinone hydrazone hydrochloride monohydrate
MS: mass spectrometry
RG: rapiGest SF surfactant
*TvL*: *Trametes versicolor* laccase
UHQ: ultrapure water
Y: tyrosine
