# Supplementary material for: Comparative Characterization of Oxidative Enzymes for Arabinoxylan and Protein Cross-Linking via Ferulic Acid and Tyrosine in Model Systems
Source: J Agric Food Chem. 2025 Dec 7;73(50):32183–94. doi: 10.1021/acs.jafc.5c09766 (PMC12715794; doi:10.1021/acs.jafc.5c09766)
Supplement: Supplementary file 1 [file jf5c09766_si_001.pdf]

SUPPLEMENTARY DATA

Comparative Characterization of Oxidative Enzymes for Arabinoxylan and Protein Cross-Linking via Ferulic Acid and Tyrosine in Model Systems

Katharina Hoefler[†,‡], Ulrich Sukop[‡], Elisabeth Reiter[†], Denisse Bender[‡], Mario Jekle[‖], Patrik Roch[†,§], Margit Cichna-Markl[§], Stefano D'Amico[†,*], Regine Schoenlechner[‡]

[†] AGES – Austrian Agency for Health and Food Safety, Institute for Animal Nutrition and Feed, Spargelfeldstraße 191, 1220 Vienna, Austria

[‡] BOKU University, Department of Biotechnology and Food Science, Muthgasse 18, 1190 Vienna, Austria

[‖] University of Hohenheim – Department of Plant-based Foods, Garbenstraße 25, 70599 Stuttgart, Germany

[§] University of Vienna – Faculty of Chemistry, Department of Analytical Chemistry, Währinger Straße 38, 1090 Vienna, Austria

* Corresponding author: Stefano.d-amico@ages.at

Table S1: Chromatographic gradient conditions for RP-HPLC measurements at a constant flow rate of 0.7 mL / min with a C-18 column (100 mm x 3 mm) at 40 °C with eluent A = 10 mM KH_2PO_4 (pH 2.0).

**Time (min)**	**%B (KH_2PO_4 with 25% ACN, pH 2.0)**	**%C (50% ACN)**
0	0	0
4	0	0
5	100	0
12	100	0
13	0	0
16	0	100
17	0	100
19	0	0

Table S2: Molar extinction coefficients (ε) of ABTS and hydroxytyrosol (HTyr)-MBTH at pH 4 – 6.5 (0.5 pH-unit steps) determined by linear regression of eight different concentrations by triplicate measurements with corresponding R^2.

**pH**	**ABTS**		**HTyr-MBTH**	
	**ε [L* mol^{-1} * cm^{-1}]**	**R^2**	**ε [L * mol^{-1} * cm^{-1}]**	**R^2**
4	33,244	0.999	10,604	0.966
4.5	34,117	0.998	13,105	0.998
5	31,514	0.998	12,062	0.999
5.5	30,827	0.999	14,545	0.995
6	30,971	0.999	16,988	0.952
6.5	35,989	0.889	13,551	0.995
Mean ± SD	32,777 ± 2,000		13,476 ± 2,200	

With MBTH = 3-Methyl-2-benzothiazolinone hydrazone hydrochloride monohydrate; ABTS = 2,2'-Azino-bis(3-ethylbenzothiazoline-6-sulfonic acid) diammonium salt

Table S3: Protein content of *TvL, HrP, AbT* and *AnG* determined via Bradford assay with BSA as standard. Values are given as mean ± SD (n = 4).

**Enzyme**	*TvL*	*HrP*	*AbT*	*AnG*
**Protein concentration [%]**	0.22 ± 0.02	47.10 ± 0.40	16.54 ± 0.07	44.7 ± 1.3

With *AbT* = *Agaricus bisporus* tyrosinase; *TvL* = *Trametes versicolor* laccase; *HrP* = *Horseradish* peroxidase; *AnG* = *Aspergillus niger* glucose oxidase

Table S4: Raw data of the effect of different pH values (4 – 6.5; 0.5 pH-unit steps) on the specific enzyme activity of *TvL, HrP, AbT* and *AnG* in McIlvaine buffer at 30 °C in a reaction volume of 0.34 mL as illustrated in Figure 2. Results are given as mean ± SD (n=3).

**pH**	**Substrate**	**Specific enzyme activity [U / mg]**[a]			
		TvL	***HrP***	***AbT***	***AnG***
4	ABTS*	650 ± 80	420 ± 70	NA	80 ± 5
4.5		350 ± 40	250 ± 40		168 ± 14
5		230 ± 20	156 ± 9		158 ± 9
5.5		166 ± 14	84 ± 13		116 ± 7
6		68 ± 3	52 ± 9		120 ± 20
6.5		9.0 ± 1.0	25 ± 2		138 ± 11
4	HTyr-MBTH**	10.0 ± 0.4	62 ± 3	4.24 ± 0.02	NA
4.5		14.0 ± 0.2	119 ± 12	11 ± 3	
5		8.7 ± 0.2	260 ± 30	25.2 ± 0.3	
5.5		7.0 ± 0.1	440 ± 60	33.9 ± 1.4	
6		3.8 ± 0.9	600 ± 40	15.5 ± 1.1	
6.5		1.8 ± 0.1	740 ± 18	32.1 ± 0.7	

With *AbT* = *Agaricus bisporus* tyrosinase; *TvL* = *Trametes versicolor* laccase; *HrP* = *Horseradish* peroxidase; *AnG* = *Aspergillus niger* glucose oxidase; HTyr = Hydroxytyrosole, MBTH = 3-Methyl-2-benzothiazolinone hydrazone hydrochloride monohydrate; ABTS = 2,2'-Azino-bis(3-ethylbenzothiazoline-6-sulfonic acid) diammonium salt

[a]Enzyme concentrations were adapted till absorbance change of 0.1 – 0.2 per minute was reached. Specific enzyme activity was calculated based on the protein content (Table S3).

*ABTS assay (*TvL, HrP, AnG*) contained 1 mM ABTS and in the case of *HrP* 1 mM H_2O_2 and in the case of *AnG* 16 mM glucose and excess of *HrP* and absorption change at 415 nm was monitored.

**Hydroxytyrosol (HTyr)-MBTH assay (*TvL, HrP, AbT*) contained 1 mM HTyr, excess of MBTH and 2% DMF and absorbance change at 500 nm was monitored.

Table S5: Raw data of the effect of different temperatures (25 – 50 °C; 5 °C steps) on the specific enzyme activity of *TvL*, *HrP* and *AnG* in McIlvaine buffer at pH 4.5 and for *AbT* at pH 6.5 in a reaction volume of 0.34 mL as illustrated in Figure 3. Results are given as mean + SD (n=3).

**T [°C]**	**Specific enzyme activity [U / mg][a]**											
	TvL*			***HrP****			***AbT****			***AnG*****		
25	650	±	80	420	±	70	32.1	±	0.7	168	±	14
30	680	±	40	441	±	90	25	±	6	215	±	13
35	730	±	40	200	±	40	20.0	±	1.5	215	±	19
40	666	±	40	98	±	20	14.2	±	0.9	216	±	4
50	620	±	30	74	±	15	14.2	±	0.7	246	±	7

With *AbT* = *Agaricus bisporus* tyrosinase; *TvL* = *Trametes versicolor* laccase; *HrP* = *Horseradish* peroxidase; *AnG* = *Aspergillus niger* glucose oxidase

[a]Enzyme concentrations were adapted till absorbance change of 0.1 – 0.2 per minute was reached. Specific enzyme activity was calculated based on the protein content (Table S3).

*ABTS assay (*TvL*, *HrP*, *AnG*) contained 1 mM ABTS and in the case of *HrP* 1 mM H_2O_2 and in the case of *AnG* 16 mM glucose and excess of *HrP* and absorption change at 415 nm was monitored.

**Hydroxytyrosol (HTyr)-MBTH assay (*AbT*) contained 1 mM HTyr, excess of MBTH and 2% DMF and absorbance change at 500 nm was monitored.

Table S6: Volumetric enzyme activities [U / mL] at different substrate concentrations, on which basis K_m (mM) and v_{max} (U / mL and U / mg) values of *AbT, TvL, HrP* and *AnG* for HTyr-MBTH and ABTS assays were calculated (Table 1). Results are given as mean ± SD (n=3).

Enzyme	pH	Temperature [°C]	Substrate	c [mM]	Volumetric enzyme activity [U / mL]
TvL	pH 4	35 °C	ABTS	0.473	2.4 ± 0.8
				0.315	2.4 ± 0.4
				0.158	2.4 ± 0.3
				0.126	1.9 ± 0.3
				0.095	1.9 ± 0.3
				0.063	1.6 ± 0.3
HrP	pH 4	25 °C	ABTS	0.090	72 ± 13
				0.079	66 ± 5
				0.063	70 ± 12
				0.047	73 ± 9
				0.032	64 ± 4
				0.016	45 ± 9
AnG	pH 4.5	40 °C	ABTS	0.129	290 ± 50
				0.066	311 ± 60
				0.038	326 ± 13
				0.020	264 ± 19
				0.013	220 ± 30
				0.007	174 ± 18
TvL	pH 4.5	35 °C	HTyr-MBTH	0.347	0.044 ± 0.003
				0.294	0.045 ± 0.003
				0.250	0.044 ± 0.004
				0.206	0.042 ± 0.002
				0.147	0.037 ± 0.002
				0.103	0.030 ± 0.001
HrP	pH 6.5	25 °C	HTyr-MBTH	0.298	547 ± 16
				0.265	510 ± 30
				0.235	469 ± 12
				0.206	450 ± 30
				0.162	288 ± 12
				0.118	220 ± 20
AbT	pH 6.5	25 °C	HTyr-MBTH	0.294	1.48 ± 0.05
				0.206	1.93 ± 0.04
				0.176	1.37 ± 0.11
				0.147	1.20 ± 0.05
				0.118	1.20 ± 0.08
				0.074	1.07 ± 0.09

With *AbT* = *Agaricus bisporus* tyrosinase; *TvL* = *Trametes versicolor* laccase; *HrP* = *Horseradish* peroxidase; *AnG* = *Aspergillus niger* glucose oxidase; HTyr = Hydroxytyrosole, MBTH = 3-Methyl-2-benzothiazolinone hydrazone hydrochloride monohydrate; ABTS = 2,2'-Azino-bis(3-ethylbenzothiazoline-6-sulfonic acid) diammonium salt

Table S7: Volumetric enzyme activities [U / mL] at different substrate concentrations, on which basis K_m (mM) and v_{max} (U / mL and U / mg) values of *AbT, TvL, HrP* and *AnG* for ferulic acid (FA) or tyrosine (Y) were calculated (Table 2). Results are given as mean ± SD (n=3).

**Enzyme**	**pH**	**Temperature [°C]**	**Substrate**	**c [mM]**	**Volumetric enzyme activity [U / mL]**
TvL	pH 4.5	30 °C	FA	0.118	0.68 ± 0.03
				0.100	0.61 ± 0.05
				0.082	0.57 ± 0.05
				0.071	0.50 ± 0.03
				0.047	0.42 ± 0.07
				0.029	0.366 ± 0.014
HrP	pH 4.5	30 °C	FA	0.118	350 ± 30
				0.100	250 ± 20
				0.082	209 ± 13
				0.071	190 ± 30
				0.059	210 ± 19
				0.041	120 ± 20
TvL + *AbT*	pH 4.5	30 °C	FA	0.176	0.61 ± 0.04
				0.141	0.54 ± 0.03
				0.118	0.53 ± 0.05
				0.094	0.46 ± 0.05
				0.071	0.38 ± 0.05
				0.047	0.301 ± 0.012
AbT +*TvL*	pH 4.5	30 °C	FA	0.176	0.142 ± 0.010
				0.141	0.128 ± 0.007
				0.118	0.123 ± 0.012
				0.094	0.108 ± 0.011
				0.071	0.088 ± 0.012
				0.047	0.070 ± 0.003
HrP + *AnG*	pH 4.5	30 °C	FA	0.141	1.23 ± 0.07
				0.118	1.30 ± 0.04
				0.100	1.07 ± 0.04
				0.082	0.94 ± 0.03
				0.059	1.02 ± 0.03
				0.035	0.73 ± 0.04
AbT	pH 6.5	30 °C	Y	0.195	0.064 ± 0.008
				0.165	0.057 ± 0.007
				0.141	0.052 ± 0.008
				0.118	0.043 ± 0.002
				0.094	0.031 ± 0.004
				0.071	0.025 ± 0.002
TvL + *AbT*	pH 6.5	30 °C	Y	0.297	0.089 ± 0.005
				0.265	0.086 ± 0.006
				0.235	0.080 ± 0.007
				0.191	0.072 ± 0.004
				0.147	0.053 ± 0.003
				0.088	0.037 ± 0.002

AbT + *TvL*	pH 6.5	30 °C	Y	0.297	0.045 ± 0.003
				0.265	0.043 ± 0.003
				0.235	0.040 ± 0.003
				0.191	0.036 ± 0.002
				0.147	0.027 ± 0.001
				0.088	0.019 ± 0.001

With *AbT* = *Agaricus bisporus* tyrosinase; *TvL* = *Trametes versicolor* laccase; *HrP* = *Horseradish* peroxidase; *AnG* = *Aspergillus niger* glucose oxidase; FA = Ferulic acide; Y = Tyrosine

Table S8: Raw data on the effect of different buffers (McIlvaine, AMAC, AMBIC) and RapiGest SF surfactant (RG) as solubilization agent at different concentrations on the volumetric enzyme activity of *TvL*, *HrP* and *AbT* at 30 °C in a reaction volume of 1.5 mL containing 100 µM ferulic acid (*TvL, HrP*) or 330 µM tyrosine (*AbT*) with 0.03 U / mL enzyme for ferulic acid and 0.01 U / mL for tyrosine as illustrated in Figure 4. Results are shown as mean + SD (n=3).

**System**	**Volumetric enzyme activity [U / mL]**		
	TvL	***HrP***	***AbT***
McIlvaine pH 4.5	0.47 ± 0.04	257 ± 14	NA
0.1 M AMAC	0.51 ± 0.04	251 ± 7	204 ± 13
0.1 M AMAC + 0.1 % RG	0.36 ± 0.03	231 ± 10	96 ± 3
0.1 M AMAC + 0.05 % RG	0.47 ± 0.03	225 ± 7	210 ± 4
McIlvaine pH 6.5	NA	144 ± 6	219 ± 13
0.1 M AMBIC pH 6.5	NA	21 ± 4	182 ± 6
0.05 M AMBIC pH 6.5	NA	NA	218 ± 10
0.025 M AMBIC pH 6.5	NA	33 ± 3	NA

With *AbT* = *Agaricus bisporus* tyrosinase; *TvL* = *Trametes versicolor* laccase; *HrP* = *Horseradish* peroxidase; AMAC = Ammonium acetate; AMBIC = Ammonium bicarbonate

Table S9: Raw data of the conversion of tyrosine (Y) and ferulic acid (FA) over time (0 h (immediately after enzyme addition), 0.25, 0.5, 1, 2, 4, 6 and 24 h) in ratio 1:1 and 1:5 (FA:Y) for *TvL*, *HrP*, *TvL* with *AbT* and *HrP* with *AnG* at pH 4.5 in 0.1 M AMAC buffer and for *HrP* and *AbT* at pH 6.5 in 0.1 M AMBIC buffer in a 3 mL reaction volume containing 0.03 U / mL enzyme. Results are shown as mean ± SD (n=3), with * indicating significant differences after 24 h ($p \leq 0.05$).

Sample	0 h	0.25 h	0.5 h	1 h	2 h	4 h	6 h	24 h
*TvL*_1:1_Y	2.63 ± 0.07	2.98 ± 0.10*	2.862 ± 0.002*	3.04 ± 0.01*	3.05 ± 0.12*	3.26 ± 0.14*	2.99 ± 0.12*	2.61 ± 0.11
*TvL*_1:1_FA	1.8 ± 0.3	0.30 ± 0.02*	0.239 ± 0.012*	<LOQ	<LOQ	<LOQ	<LOQ	<LOQ
*TvL*_1:5_Y	14.4 ± 0.3	15.6 ± 0.8	14.3 ± 0.9	15.7 ± 0.4	15.8 ± 0.8	16.5 ± 1.2*	17.4 ± 0.6*	14.60 ± 0.17
*TvL*_1:5_FA	1.4 ± 0.2	0.27 ± 0.03*	0.28 ± 0.03*	<LOQ	<LOQ	<LOQ	<LOQ	<LOQ
*HrP*_pH4.5_1:1_Y	2.93 ± 0.13	3.3 ± 0.4	3.2 ± 0.3	2.68 ± 0.10	3.5 ± 0.3	3.35 ± 0.05	3.5 ± 0.6	3.098 ± 0.009
*HrP*_pH4.5_1:1_FA	0.6 ± 0.2	0.31 ± 0.07*	0.30 ± 0.04*	<LOQ	<LOQ	<LOQ	<LOQ	<LOQ
*HrP*_pH4.5_1:5_Y	15.8 ± 0.7	16.1 ± 1.5	17.3 ± 0.8	16.3 ± 0.6	16.3 ± 0.9	19.09 ± 0.02**	15.6 ± 1.1	16.3 ± 0.8
*HrP*_pH4.5_1:5_FA	0.35 ± 0.08	0.29 ± 0.06	0.24 ± 0.02*	<LOQ	<LOQ	<LOQ	<LOQ	<LOQ
*HrP*_pH6.5_1:1_Y	3.0 ± 0.2	3.4 ± 0.2	3.4 ± 0.3	3.09 ± 0.05	3.7 ± 0.6	3.8 ± 0.8	3.90 ± 0.15	3.01 ± 0.15
*HrP*_pH6.5_1:1_FA	0.74 ± 0.10	0.46 ± 0.04*	0.32 ± 0.04*	0.31 ± 0.08*	<LOQ	<LOQ	<LOQ	<LOQ
*HrP*_pH6.5_1:5_Y	16.5 ± 0.9	17.0 ± 1.1	17.2 ± 1.5	15.5 ± 1.9	17.9 ± 1.0	17 ± 3	18 ± 3	15.9 ± 1.1
*HrP*_pH6.5_1:5_FA	0.25 ± 0.02	0.35 ± 0.09*	0.555 ± 0.007*	<LOQ	<LOQ	<LOQ	<LOQ	<LOQ
*AbT*_1:1_Y	2.6 ± 0.2	3.4 ± 0.3	3.2 ± 0.7	3.4 ± 0.9	3.7 ± 0.8	3.4 ± 0.7	3.6 ± 0.5	2.64 ± 0.09
*AbT*_1:1_FA	2.9 ± 0.3	4.07 ± 0.12*	3.5 ± 0.4	3.2 ± 0.9	2.1 ± 0.5	0.9 ± 0.3*	0.66 ± 0.16*	<LOQ
*AbT*_1:5_Y	16.3 ± 0.5	12.8 ± 1.0*	8 ± 2*	2.5 ± 0.4*	<LOQ	<LOQ	<LOQ	<LOQ
*AbT*_1:5_FA	3.2 ± 0.3	4.4 ± 0.6	4.4 ± 0.5	3.8 ± 0.9	3.1 ± 0.4	3.8 ± 0.9	3.5 ± 0.9	1.17 ± 0.13*
*AbT+TvL*_1:1_Y	2.9 ± 0.4	3.4 ± 0.4	3.3 ± 0.2	3.3 ± 0.3	3.16 ± 0.18	3.4 ± 0.4	3.1 ± 0.2	3.24 ± 0.19
*AbT+TvL*_1:1_FA	0.48 ± 0.06	0.32 ± 0.06*	0.45 ± 0.11	<LOQ	<LOQ	<LOQ	<LOQ	<LOQ
*AbT+TvL*_1:5_Y	16.0 ± 0.6	16.0 ± 1.5	16.7 ± 1.1	18 ± 3	17.4 ± 1.0	17 ± 2	16.8 ± 1.7	15.8 ± 0.5
*AbT+TvL*_1:5_FA	2.4 ± 1.2	0.40 ± 0.12*	0.32 ± 0.13*	<LOQ	<LOQ	<LOQ	<LOQ	<LOQ
*HrP+AnG*_1:1_Y	3.1 ± 0.2	3.0 ± 0.2*	2.1 ± 0.4*	1.4 ± 0.3*	<LOQ	<LOQ	<LOQ	<LOQ
*HrP+AnG*_1:1_FA	0.5 ± 0.4	<LOQ	<LOQ	<LOQ	<LOQ	<LOQ	<LOQ	<LOQ
*HrP+AnG*_1:5_Y	17.0 ± 0.7	14.9 ± 1.4*	11.7 ± 1.7*	8.3 ± 0.9*	9 ± 2*	3.2 ± 1.5*	<LOQ	<LOQ
*HrP+AnG*_1:5_FA	0.8 ± 0.5	<LOQ	<LOQ	<LOQ	<LOQ	<LOQ	<LOQ	<LOQ
AMAC_1:1_Control_Y	2.3 ± 0.2	NA	NA	NA	NA	NA	NA	2.2 ± 0.4
AMAC+H_2O_2_1:1_Control_Y	2.6 ± 0.4	NA	NA	NA	NA	NA	NA	2.3 ± 0.2
AMAC_1:5_Control_Y	14.1 ± 1.5	NA	NA	NA	NA	NA	NA	13.1 ± 0.9
AMAC+H_2O_2_1:5_Control_Y	12.8 ± 1.6	NA	NA	NA	NA	NA	NA	12.9 ± 0.4
AMAC_1:1_Control_FA	5.4 ± 0.4	NA	NA	NA	NA	NA	NA	4.6 ± 0.2*
AMAC+H_2O_2_1:1_Control_FA	5.1 ± 0.7	NA	NA	NA	NA	NA	NA	3.6 ± 0.6*

AMAC_1:5_Control_FA	5.1 ± 0.4	NA	NA	NA	NA	NA	NA	4.5 ± 0.3
AMAC+H_2O_2_1:5_Control_FA	5.0 ± 0.8	NA	NA	NA	NA	NA	NA	4.8 ± 1.1
AMBIC_1:1_Control_Y	2.142 ± 0.06	NA	NA	NA	NA	NA	NA	2.42 ± 0.3
AMBIC+H2O2_1:1_Control_Y	2.6 ± 0.6	NA	NA	NA	NA	NA	NA	2.7 ± 0.6
AMBIC_1:5_Control_Y	13.7 ± 0.2	NA	NA	NA	NA	NA	NA	12.7 ± 0.7
AMBIC+H_2O_2_1:5_Control_Y	15.4 ± 3.8	NA	NA	NA	NA	NA	NA	16.4 ± 2.2
AMBIC_1:1_Control_FA	4.86 ± 0.07	NA	NA	NA	NA	NA	NA	2.62 ± 0.12*
AMBIC+H_2O_2_1:1_Control_FA	5.0 ± 1.1	NA	NA	NA	NA	NA	NA	2.9 ± 0.6*
AMBIC_1:5_Control_FA	6.1 ± 0.7	NA	NA	NA	NA	NA	NA	5.1 ± 0.6
AMBIC+H_2O_2_1:5_Control_FA	5.1 ± 1.4	NA	NA	NA	NA	NA	NA	5.0 ± 0.6

With *AbT* = *Agaricus bisporus* tyrosinase; *TvL* = *Trametes versicolor* laccase; *HrP* = *Horseradish* peroxidase; *AnG* = *Aspergillus niger* glucose oxidase; AMAC = Ammonium acetate; AMBIC = Ammonium bicarbonate; FA = Ferulic acide; Y = Tyrosine

Figure S1: UV-vis spectra of the conversion capability of the oxidative enzymes *AbT, HrP, TvL, AnG* on tyrosine (Y) and ferulic acid (FA). The absorbance changes over 60 min are shown, after incubated of the enzymes in McIlvaine buffer at pH 4.5 or 6.5 at 30 °C in a reaction volume of 0.34 mL with 2 mM ferulic acid or 0.2 mM tyrosine, respectively. With (A) is showing Y with *TvL*, (B) FA with *TvL*, (C) Y with *HrP*, (D) Fa with *HrP*, (E) Y with *AbT*, (F) FA with *AbT*, (G) Y with *AnG* and (H) FA with *AnG*.

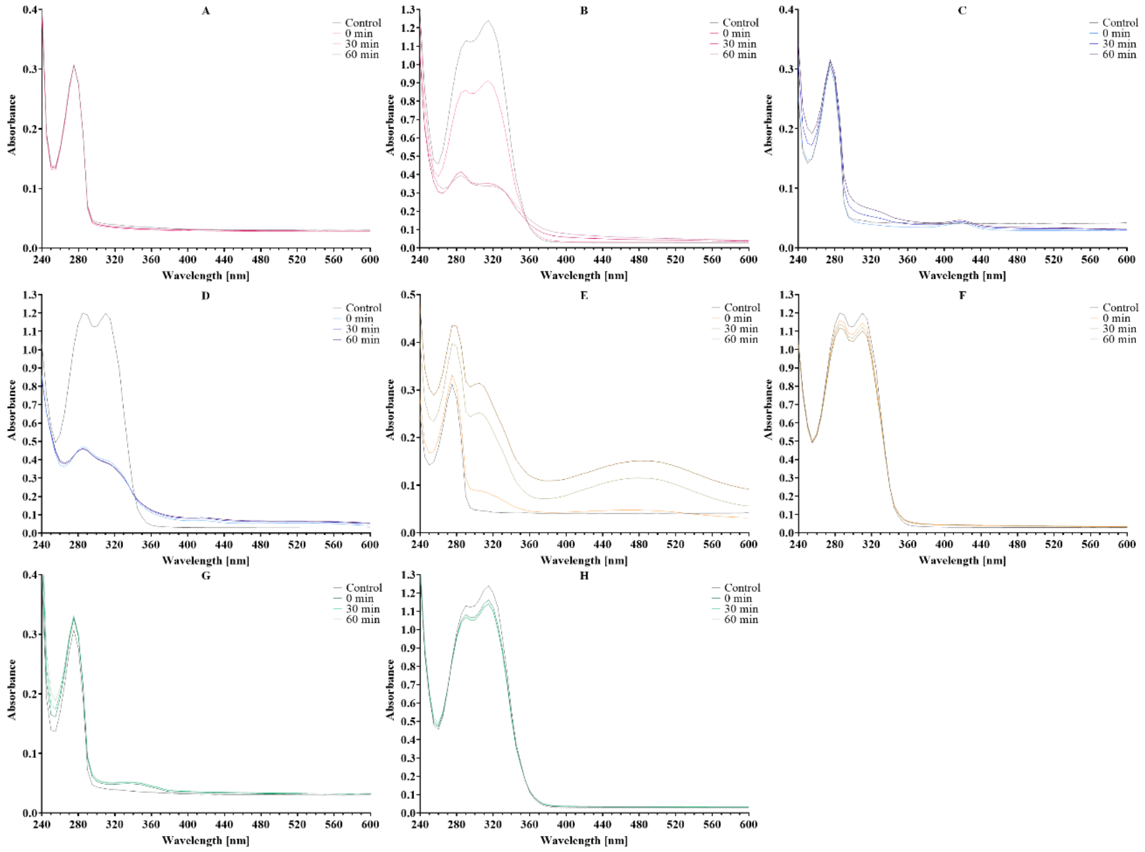

Figure S2: UV-vis spectra of the conversion capability of the oxidative enzyme combination of *AbT* with *TvL* and *HrP* with *AnG* on tyrosine (Y) and ferulic acid (FA). The absorbance changes over 5 h are shown, after incubated of the enzymes in McIlvaine buffer at pH 4.5 or 6.5 at 30 °C in a reaction volume of 0.34 mL with 2 mM ferulic acid or 0.2 mM tyrosine, respectively. With (A) is showing Y with *TvL* and *AbT* at pH 4.5, (B) Y with *TvL* and *AbT* at pH 6.5, (C) FA with *TvL* and *AbT* at pH 4.5, (D) FA with *TvL* and *AbT* at pH 6.5, (E) FA with *HrP* and *AnG* at pH 4.5 and (F) Y with *HrP* and *AnG* at pH 4.5.

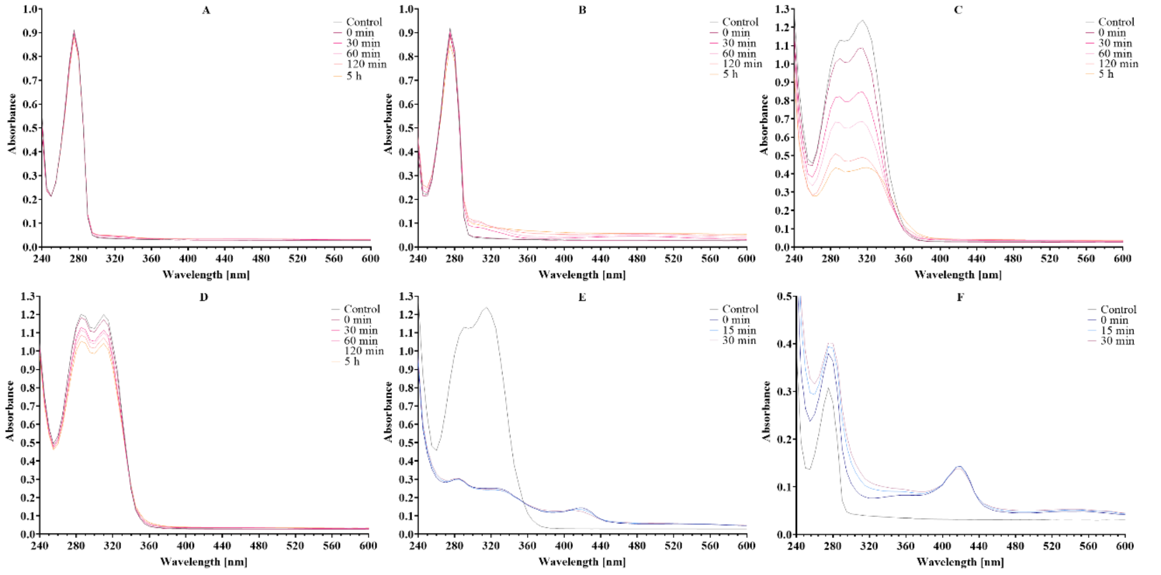

Figure S3: Calibration of ferulic acid (FA) (left) based on HPLC-DAD response at 320 nm and tyrosine (Y) (right) based on response at 280 nm. Regression equation with corresponding R^2 as function of substrate concentration to response is given, respectively. A typically chromatogram (conditions cf. Table S1) with corresponding retention time for Y and FA is illustrated in the bottom.

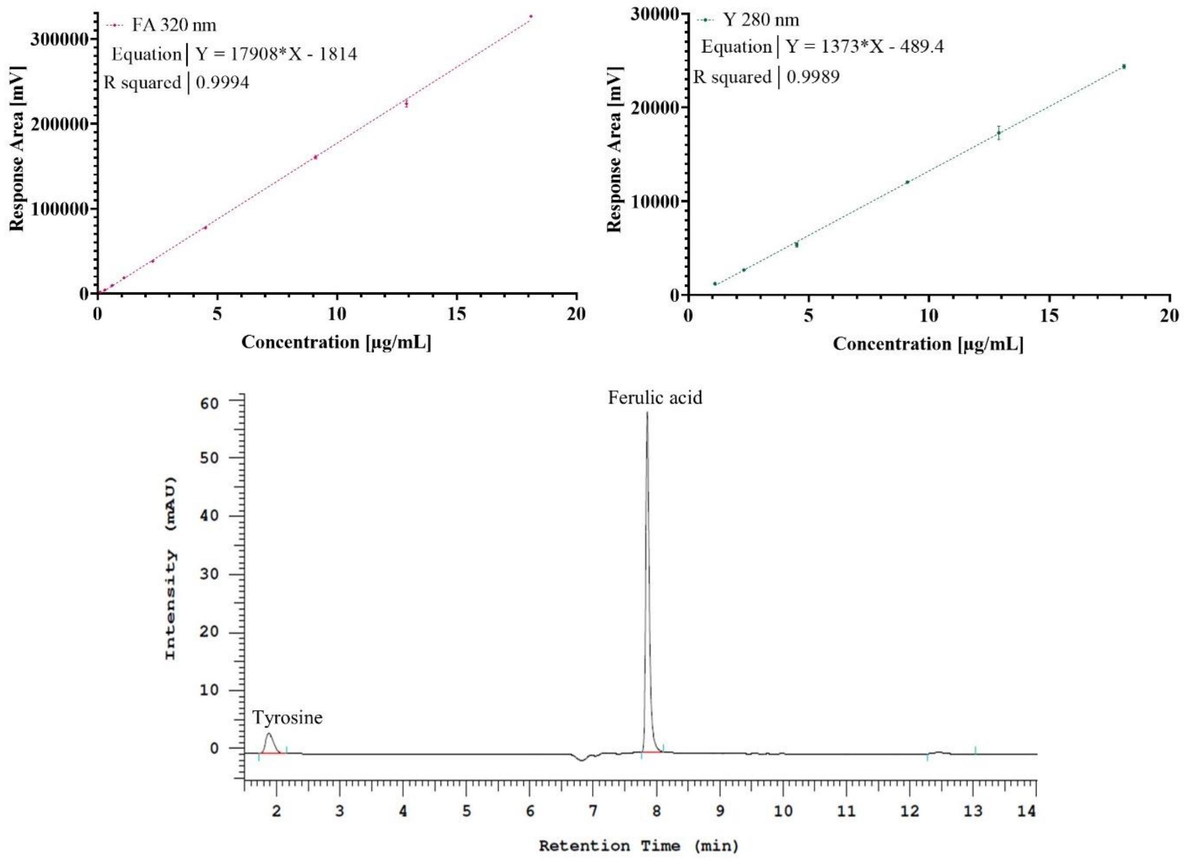

Figure S4: Selection of catalyzed reactions of *Trametes versicolor* Laccase (*TvL*), *Horseradish* Peroxidase (Hr*P*), *Aspergillus niger* glucose oxidase (*AnG*) and *Agaricus bisporus* tyrosinase (*AbT*). A, oxidation of ferulic acid (FA) (1a) bound to arabinoxylan (Ara) catalysed with *TvL* (oxygen consumption) or *HrP* (hydrogen peroxide consumption) to three possible radicals (1b–1d)[1,2]. B, oxidation of glucose (2a) catalysed by *AnG* (oxygen consumption) to gluconolactone (2b) while producing H_2O_2[3]. C, oxidation of tyrosine (3a) bound to protein (Prot) catalyzed by *HrP* or *TvL* to four possible radicals (3b–3e), and monophenol hydroxylation catalyzed by *AbT* (oxygen consumption) to catechol (3f) and diphenol oxidation to ortho-quinone (3g)[4,5]. D, possible homocross-linking reaction of FA radicals to decarboxylated dehydrodimers (4a) and further to hetero-trimeric radicals (4b)[1]. E, possible homo cross-links of tyrosine radicals to dimeric structures (5a, 5b)[4]. F, possible heterocross-linking reaction of FA and tyrosine radicals to hetero-dimeric (6a, 6b) or -trimeric (6c) structures[6]. G, *AbT* catalyzed quinone product reaction to homocross-link (7a) or further nonenzymatic reaction with other amino acid residues via Schiff's base reaction (7b) or Michaelis addition (7c,7d)[5,7]. H, possible heterocross-link reaction of *AbT* catalyzed quinone product with FA to form hetero-adducts (8a)[2].

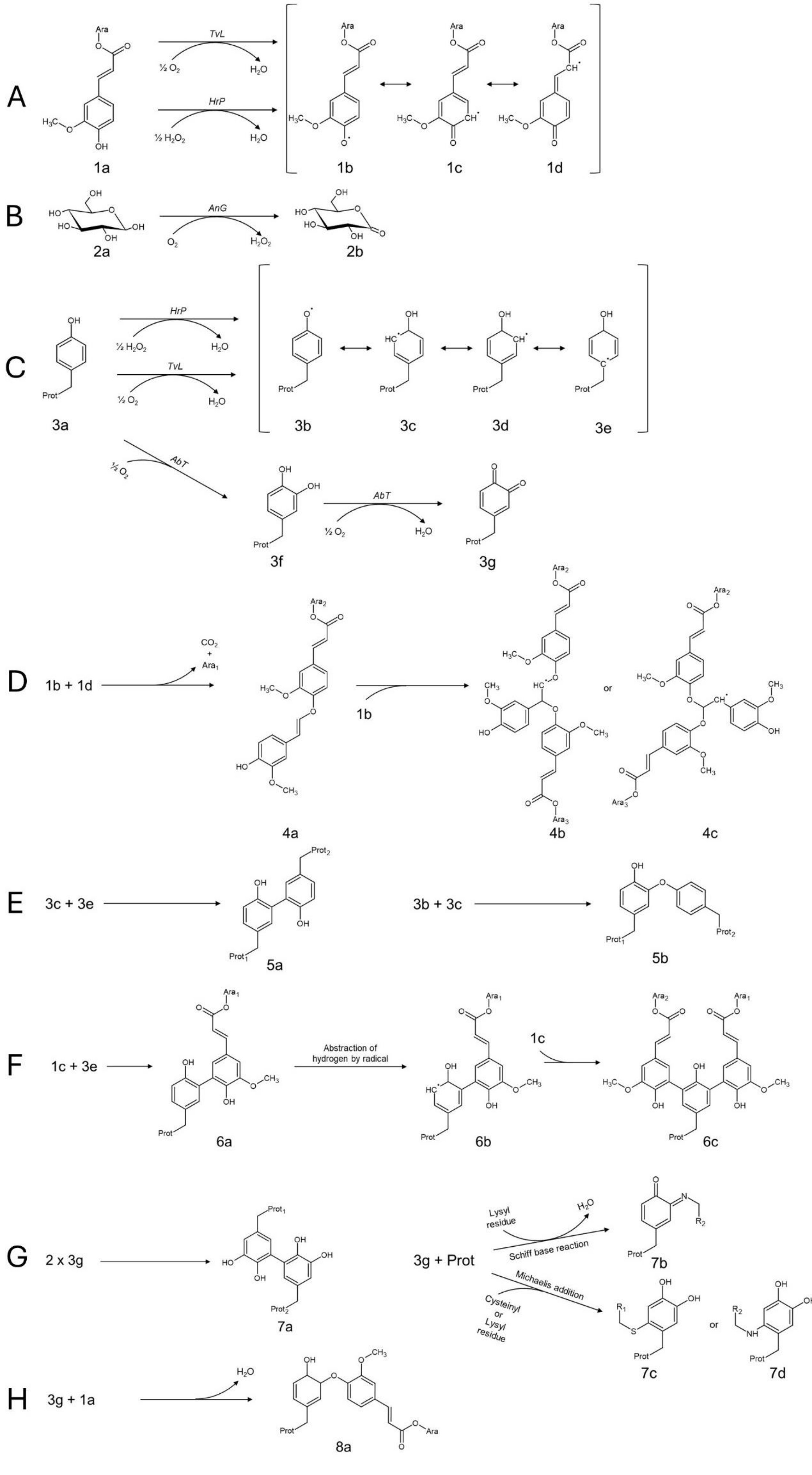
A
Ara
TvL
½ O2
H2O
HrP
½ H2O2
H3C
OH
1a
1b
1c
1d
B
AnG
O2
H2O2
2a
2b
C
HrP
TvL
AbT
Prot
3a
3b
3c
3d
3e
3f
3g
D
1b + 1d
CO2
+
Ara1
Ara2
Ara3
1b
or
4a
4b
4c
E
3c + 3e
3b + 3c
Prot1
Prot2
5a
5b
F
1c + 3e
Abstraction of
hydrogen by radical
1c
6a
6b
6c
G
2 x 3g
3g + Prot
Lysyl
residue
Schiff base reaction
Michaelis addition
Cysteinyl
or
Lysyl
residue
R1
R2
or
7a
7b
7c
7d
H
3g + 1a
8a

References

(1) Oudgenoeg, G.; Dirksen, E.; Ingemann, S.; Hilhorst, R.; Gruppen, H.; Boeriu, C. G.; Piersma, S. R.; van Berkel, W. J.; Laane, C.; Voragen, A. G. Horseradish Peroxidase-catalyzed Oligomerization of Ferulic Acid on a Template of a Tyrosine-containing Tripeptide. *Journal of Biological Chemistry* **2002**, *277* (24), 21332–21340. DOI: 10.1074/jbc.M201679200

(2) Selinheimo, E.; Lampila, P.; Mattinen, M.-L.; Buchert, J. Formation of protein-oligosaccharide conjugates by laccase and tyrosinase. *Journal of Agricultural and Food Chemistry* **2008**, *56* (9), 3118–3128. DOI: 10.1021/jf0730791

(3) Meyer, M.; Wohlfahrt, G.; Knäblein, J.; Schomburg, D. Aspects of the mechanism of catalysis of glucose oxidase: A docking, molecular mechanics and quantum chemical study. *Journal of Computer-Aided Molecular Design* **1998**, *12*, 425–440. DOI: 10.1023/A:1008020124326

(4) Mattinen, M.-L.; Kruus, K.; Buchert, J.; Nielsen, J. H.; Andersen, H. J.; Steffensen, C. L. Laccase-catalyzed polymerization of tyrosine-containing peptides. *The FEBS Journal* **2005**, *272* (14), 3640–3650. DOI: https://doi.org/10.1111/j.1742-4658.2005.04786.x.

(5) Zeeb, B.; Fischer, L.; Weiss, J. Stabilization of food dispersions by enzymes. *Food & Function* **2014**, *5* (2), 198–213. DOI: 10.1039/C3FO60499C

(6) Oudgenoeg, G.; Hilhorst, R.; Piersma, S.; Boeriu, C.; Gruppen, H.; Hessing, M.; Voragen, A.; Laane, C. Peroxidase-Mediated Cross-Linking of a Tyrosine-Containing Peptide with Ferulic Acid. *Journal of Agricultural and Food Chemistry* **2001**, *49*, 2503–2510. DOI: 10.1021/jf000906o

(7) Li, X.; Li, S.; Liang, X.; McClements, D. J.; Liu, X.; Liu, F. Applications of oxidases in modification of food molecules and colloidal systems: Laccase, peroxidase and tyrosinase. *Trends in Food Science & Technology* **2020**, *103*, 78–93. DOI: 10.1016/j.tifs.2020.06.014
